# Genome-wide identification and characterisation of human DNA replication origins by initiation site sequencing (ini-seq)

**DOI:** 10.1093/nar/gkw760

**Published:** 2016-09-01

**Authors:** Alexander R. Langley, Stefan Gräf, James C. Smith, Torsten Krude

**Affiliations:** 1 Francis Crick Institute, Mill Hill Laboratory, The Ridgeway, London NW7 1AA, UK; 2 Department of Medicine, University of Cambridge, Cambridge CB2 0QQ, UK; 3 Department of Haematology, University of Cambridge, Cambridge CB2 0PT, UK; 4 Department of Zoology, University of Cambridge, Downing Street, Cambridge CB2 3EJ, UK

## Abstract

Next-generation sequencing has enabled the genome-wide identification of human DNA replication origins. However, different approaches to mapping replication origins, namely (i) sequencing isolated small nascent DNA strands (SNS-seq); (ii) sequencing replication bubbles (bubble-seq) and (iii) sequencing Okazaki fragments (OK-seq), show only limited concordance. To address this controversy, we describe here an independent high-resolution origin mapping technique that we call initiation site sequencing (ini-seq). In this approach, newly replicated DNA is directly labelled with digoxigenin-dUTP near the sites of its initiation in a cell-free system. The labelled DNA is then immunoprecipitated and genomic locations are determined by DNA sequencing. Using this technique we identify >25,000 discrete origin sites at sub-kilobase resolution on the human genome, with high concordance between biological replicates. Most activated origins identified by ini-seq are found at transcriptional start sites and contain G-quadruplex (G4) motifs. They tend to cluster in early-replicating domains, providing a correlation between early replication timing and local density of activated origins. Origins identified by ini-seq show highest concordance with sites identified by SNS-seq, followed by OK-seq and bubble-seq. Furthermore, germline origins identified by positive nucleotide distribution skew jumps overlap with origins identified by ini-seq and OK-seq more frequently and more specifically than do sites identified by either SNS-seq or bubble-seq.

## INTRODUCTION

S phase of the human cell cycle takes 8–10 h. To achieve complete genome duplication during this time, DNA replication is initiated at about 30,000–50,000 independent sites (1,2). The identification of these DNA replication origins on the genome is important for our understanding of the regulation of chromosomal DNA replication.

Early mapping experiments in human cells have identified only few individual replication origins (3), which are localised in the vicinity of promoters such as those of the *MYC, TOP1, MCM4* and *beta-globin* genes (4–7), and in the case of the ‘laminB2 origin’, in the region comprising the terminus of the *LMNB2* gene and the promoter of the short *TIMM13* gene (8,9). By transplanting them to different genomic or plasmid-borne contexts and observing origin activity at the new locations, the *MYC, beta-globin* and *laminB2* origins have also been shown to have replicator function (10–13).

The use of next-generation DNA sequencing has now led to the discovery of tens of thousands of potential replication origins in the human genome. Three independent approaches have been used that exploit the direct identification of DNA replication initiation intermediates.

The first approach builds on the isolation of short nascent DNA single strands, which are synthesised as initiation intermediates with a 5′ RNA primer on the leading strand. Nascent strands are isolated from total DNA by heat denaturation and subsequently separated from shorter Okazaki fragments by size fractionation (14). An improvement of the isolation protocol involves the degradation by lambda exonuclease of any contaminating DNA fragments without a 5′ RNA primer (15). Next-generation sequencing of these isolated short nascent strands (SNS-seq) has identified some 50,000–250,000 potential initiation sites on the human genome (16–19). These genome-wide SNS-seq studies have extended and consolidated earlier microarray hybridisation data (20–22), consistently showing that active origin sites often correlate with transcription start sites (TSS) and are located in GC-rich regions, near CpG islands and G-quadruplex (G4) sequence motifs.

A second approach is based on the sequencing of DNA replication bubbles (23). Replication bubbles are formed as early intermediates following the establishment of two divergent replication forks. To isolate such bubbles, replicating genomic DNA is fragmented by a restriction endonuclease and then embedded into gelling agarose. Replication bubbles are trapped topologically by the polymerising agarose fibres that form the gel. Linear DNA fragments and Y-shaped replication forks are electrophoresed out of the set gel while circular replication bubbles remain trapped (24). Next-generation DNA sequencing of these trapped bubbles (bubble-seq) has identified more than 100,000 sites in the human genome (23).

A third approach has used the sequencing of purified Okazaki fragments (OK-seq) for a genome-wide determination of replication fork polarity enabling the mapping of initiation and termination sites (25). This analysis identified between 5000 and 10,000 broad initiation zones of up to 150 kb that are mostly non-transcribed, often flanked by active genes, and typically contain a single but randomly located initiation event (25).

**Figure 1.**
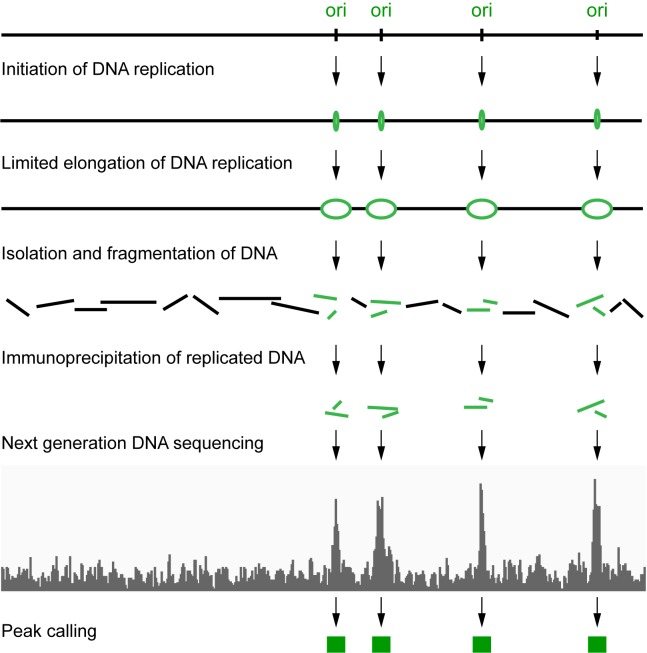
Initiation site sequencing (ini-seq). Schematic representation of the experimental approach. Green lines represent replicated and digoxigenin-labelled nascent DNA and black lines represent unreplicated double-stranded DNA. See text for details.

Unfortunately, there is limited concordance between these three approaches. Depending on peak calling parameters, only 33–65% of genomic sites identified by nascent strand and bubble trap-based approaches overlap with each other (3,19). Initiation zones determined by OK-seq overlap more frequently with the larger bubble-seq sites than with more narrowly defined SNS-seq sites (25). It is important, therefore, to design additional experimental approaches to resolve these discrepancies and to consolidate common features. To this end, we have developed the approach of DNA replication initiation site sequencing (ini-seq) (Figure 1).

Ini-seq is built on a biochemically defined cell-free system with fast and synchronous initiation of DNA replication (26–28), from which we isolate and sequence nascent DNA. Briefly, template nuclei are isolated from human cells synchronised in the late G1 phase of the cell cycle by the iron-chelating compound mimosine, which inhibits the transition of pre-replication to pre-initiation complexes at replication origins (27,29,30). DNA replication can rapidly be initiated in these template nuclei by adding a soluble extract from proliferating human cells, which contains all essential replication factors (27,31,32). We supplement the reaction with exogenous nucleoside triphosphates, including digoxigenin-dUTP as a tracer to label nascent DNA. The newly initiated replication forks have progression rates of about 300 ± 200 bp/min, which is two to five times slower than average rates observed *in vivo* because of the dilution of replication factors *in vitro* (33). The system therefore allows the synthesis of small stretches of nascent DNA emanating from activated origins under controlled biochemical conditions.

Here, we use this system to determine the locations of DNA replication initiation sites on the human genome. Use of a short incubation time permits labelling of newly replicated DNA by digoxigenin-dUTP at DNA sequences adjacent to activated replication origins. After fragmentation of genomic DNA, we isolate labelled fragments by immunoprecipitation with anti-digoxigenin antibodies, and we map their positions by next-generation sequencing and computational analysis. Our results validate earlier single-origin mapping data and provide a new and independent genome-wide dataset of >25,000 discrete locations of activated DNA replication origins on the human genome. We go on to correlate these locations with initiation zones recently identified by the sequencing of Okazaki fragments (25) and with genomic sites identified by short nascent strand (16) and bubble sequencing approaches (23), as well as with locations of origins predicted by a computational approach to be active in the germline (34,35).

Our work introduces a novel and stringent high-resolution origin mapping technique that complements and extends existing methods. Significantly, origin sites determined by ini-seq prove to be more concordant with sites identified by SNS-seq than with OK-seq or bubble-seq, and we discover that a discrete subset of replication origins identified by all available methods is present at transcription start sites that also contain G4 motifs.

## MATERIALS AND METHODS

### Cell culture, synchronisation and preparation of nuclei

Cell culture, cell synchronisation and preparation of late G1 phase nuclei from human EJ30 bladder carcinoma cells were as previously described (27,28). Cytosolic extracts of asynchronously proliferating human HeLa cells were obtained from Ipracell (Mons, Belgium).

### Immunoprecipitation of replicated DNA

Immunoprecipitation of replicated DNA is based on the cell-free DNA replication initiation assay (27). Here, 10^7^ isolated late G1 phase nuclei were incubated for 15 min under standard replication assay conditions resulting in the initiation of DNA replication and incorporation of digoxigenin-11-dUTP (Roche). Reactions were stopped by addition of ice-cold PBS and nuclei were recovered by centrifugation. Chromosomal DNA was isolated by incubation of nuclei in DNA purification buffer (10mM Tris-HCl pH8.0, 125mM NaCl, 1mM EDTA, 1% sodium lauroyl sarcosinate w/v) supplemented with 2 mg/ml proteinase K for 18 h at 55°C, followed by phenol/chloroform extraction, RNase A treatment (200 μg/ml final concentration, incubation at 37°C for 1 h) and a subsequent phenol/chloroform extraction step. The isolated chromosomal DNA was then sheared by sonication resulting in 100–1000 bp fragments. Debris was removed by centrifugation. This material constituted the ‘input’ for subsequent immunoprecipitation.

The isolated DNA preparation was then incubated for 2 hours at RT with Protein G Dynabeads (Invitrogen) coupled to anti-digoxigenin (Roche). Uncoupled beads were used as a negative control. Beads were washed six times with IP wash buffer 1 (50mM HEPES-KOH pH7.5, 500mM LiCl, 1mM EDTA, 1% Igepal CA-630 v/v, 0.7% sodium deoxycholate w/v) and then twice with IP wash buffer 2 (10 mM Tris–HCl pH8.0, 150 mM NaCl, 1 mM EDTA). Beads were transferred to a new tube and material was eluted twice for 15 min using IP elution buffer (50 mM Tris–HCl pH8.0, 1mM EDTA, 1% SDS w/v) at 65°C under agitation.

Input and immunoprecipitated DNA samples were extracted with phenol/chloroform and recovered by ethanol precipitation. Immunoprecipitated DNA fragments were analysed by quantitative PCR or next-generation DNA sequencing.

### Quantitative PCR (qPCR)

Quantitative PCR (qPCR) was performed on the LightCycler 480 II using SYBR Green I master mix according to the manufacturer's protocol (Roche). Reactions were set up in 10 μl volumes, including 0.5 μM of each primer. The sequences of the primers used are shown in Supplementary Table S1.

### Next-generation DNA Sequencing

For next-generation sequencing of DNA, libraries were generated using the Illumina ChIP-seq sample prep kit according to the manufacturer's protocol. Sequencing was performed on Illumina HiSeq2000 platform according to the manufacturer's protocol.

### Bioinformatics

#### Post-processing of sequencing reads

The quality of the raw sequencing reads was assessed with FastQC (http://www.bioinformatics.babraham.ac.uk/projects/fastqc/). Reads were then aligned against the human genome reference assembly (GRCh37) with Novoalign (Novocraft Technologies Sdn Bhd [http://www.novocraft.com], version 3.02.05) stripping potential adapter sequence from reads prior to alignment. The bam files were then further processed with Picard CleanSam (http://broadinstitute.github.io/picard/), sorted with SAMtools (36) before duplicate reads were marked with Picard. Sequencing reads aligning to problematic regions for signal detection were filtered out using the Encode Duke Excluded Regions track (https://genome.ucsc.edu/cgi-bin/hgTrackUi?g=wgEncodeMapability).

#### Peak calling

Bam files were converted to bed file format using BEDTools (http://www.ncbi.nlm.nih.gov/pubmed/20110278). Sequence tag enrichment was then detected using SICER (v1.1, http://www.ncbi.nlm.nih.gov/pubmed/19505939). In order to be able to process data aligned to GRCh37 with this algorithm the GenomeData class had to be amended accordingly. The wrapper shell scripts have been amended, so that several instances of the program could be executed in parallel. SICER was then run with and without input library using a window size of 150 bp, a redundancy threshold of 1, a fragment size of 200 bp, an effective genome fraction of 0.793, a gap size of 0 and the given respective FDR or *E*-values described in the results. The resulting peak calls were then merged if two adjacent peaks were only separated by one window (i.e. 150 bp) using bedtools intersect. The intersection between the two ini-seq peak sets was created with bedtools merge.

#### Randomisation of peak distributions

Peak distributions were randomised by using the RANDBETWEEN(*x*,*y*) function of Microsoft Excel Mac2011. For full randomisation across each chromosome, the start point of each peak in the original distribution was generated using values of *x* = 1 and *y* = (length of the respective chromosome). For randomisation within DNA replication timing windows (average width = 480,000 bp), the start point of each peak was generated by adding to the original start point the randomised value generated within *x* = -240,000 and *y* = +240,000. For both methods, the original peak length was then allocated to the randomised start point, thus generating randomised peak distributions that maintained the width and variance of the original distributions.

#### Determination of distribution overlaps

Overlaps between different peak distributions were calculated in RStudio, using the Bioconductor packages biomaRt and GenomicRanges. The minimum overlap was defined as one nucleotide. Overlaps were visualised by Venn diagrams, generated with an online tool at the Whitehead Institute for Biomedical Research (http://jura.wi.mit.edu/bioc/tools/venn.php).

#### Visualisation

Positions of mapped sequencing reads and called peaks were visualised with the Integrated Genomics Viewer version 2.3 (37,38), available at (http://www.broadinstitute.org/igv/). Screenshots were exported as svg files. Box-and-whisker plots were drawn in R software under default conditions.

### Data access

The high-throughput sequencing data from this study have been submitted to the European Nucleotide Archive (ENA; http://www.ebi.ac.uk/ena) under accession number PRJEB12207. Genomic coordinates of origin positions determined by ini-seq as the intersect of libraries IP-A and IP-B (peaks called excluding the input library) are available in bed file format as Supplementary Table S2 (for *E* = 0.1) and S3 (for *E* = 10e-5).

## RESULTS

### Immunoprecipitation of nascent DNA

Chromosomal DNA replication in nuclei isolated from synchronised human late G1 phase cells begins a few minutes after addition of an extract from proliferating cells (27,31,32). This *in vitro* initiation recapitulates the regulation of initiation in intact cells in several respects (26). First, the initiation of semiconservative DNA replication *in vitro* is dependent on the activity of S phase promoting cyclin-dependent protein kinases (27,28). Second, quantitative PCR has shown that initiation occurs at sites on the genome that are also used *in vivo*, such as the *laminB2* origin (39). Third, DNA combing and DNA fibre fluorescence microscopy have provided direct evidence for a bidirectional initiation of two replication forks moving away from their initiation sites at a rate of 300 ± 200 bp/min (33). These observations indicate that a short incubation period in this system leads to the limited synthesis of origin-proximal nascent DNA. We decided to ask whether this nascent DNA could be isolated by immunoprecipitation, sequenced, and mapped to the genome (Figure 1).

To label nascent DNA, we incubated nuclei from mimosine-arrested EJ30 cells for 15 min in a HeLa cell extract including a buffered nucleotide mix containing digoxigenin-dUTP. The EJ30 bladder carcinoma cell line shows the best synchronisation properties of all cell lines tested in our hands, leading to the highest ratio of initiation signal to elongation background noise. The incubation of these nuclei *in vitro* allows initiation to occur after a short lag phase of up to 10 min (27), followed by controlled fork progression over short distances away from the initiation sites. We observed in control experiments that 49% of the template nuclei initiated DNA replication *in vitro* over and above a contaminating background of 3.5% S phase nuclei that elongate DNA replication at pre-existing forks initiated *in vivo*, consistent with our earlier observations (27,31–33). Chromosomal DNA was then isolated and fragmented by sonication to a size of 100–1000 bp, and we were able to immunoprecipitate labelled nascent DNA in a digoxigenin-dUTP and anti-digoxigenin antibody-specific manner (Supplementary Figure S1).

**Figure 2.**
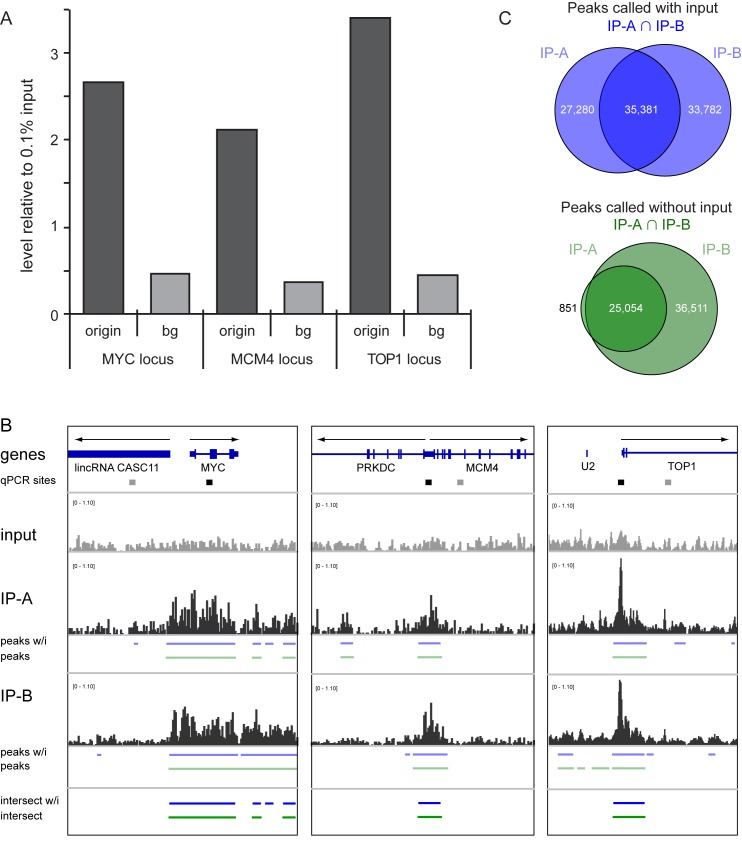
Determination of replication origin locations by initiation site sequencing (ini-seq). (**A**) Validation of immunoprecipitation by quantification of nascent DNA at established replication origins. Immunoprecipitated DNA was quantified by real-time PCR, using primer pairs located at the replication origins located near the promoters of the *MYC, MCM4* and *TOP1* gene loci (origin), and at corresponding non-origin background sites (bg). Data are expressed as proportions of 0.1% amount of input DNA, mean values of triplicate determinations are shown for one IP experiment. Sequences for the PCR primer pairs are in Supplementary Table S1, and their genomic locations are mapped in panel B. (**B**) Verification of origin positions by initiation site sequencing (ini-seq). Positions of sequencing reads are visualised on the integrated genome viewer (IGV) for total input control DNA (input) and two independent biological replicates of immunoprecipitated nascent DNA (IP-A and IP-B). Peaks of nascent DNA enrichment were called by SICER, either by integrating the input DNA (w/i; blue bars), or not doing so (green bars). The areas of peaks present in both IP-A and IP-B (i.e. the intersect) are shown for both peak-calling strategies as indicated. Chromosomal 25kb regions are shown for the *MYC* locus (chromosome 8: 128.735–128.76Mb), the *MCM4* locus (chromosome 8: 48.86-48.885Mb), and the *TOP1* locus (chromosome 20: 39.648–39.673 Mb). Positions of reference genes (blue), transcription directionalities (black arrows), and primer sites used for qPCR analyses (black, origin sites; grey, background sites) are indicated. (**C**) Venn diagrams of overlap between enrichment peaks called by two modes of SICER in the human genome for libraries A and B (IP A and IP B). Overall counts of peaks are indicated.

We used quantitative real time PCR to ask whether immunoprecipitated DNA is enriched for sequences around established replication origins near the promoters of the *MYC, MCM4* and *TOP1* genes (6,7,40). Immunoprecipitated DNA proved to be enriched at these origin sites ∼5–10 times above the background detected at non-origin control sites in DNA prepared from EJ30 cell nuclei (Figure 2A) and also from HeLa S3 cell nuclei (Supplementary Figure S2), thus validating the immunoprecipitation method.

### Origin mapping by deep sequencing of immunoprecipitated nascent DNA

We went on to map human DNA replication origins on a genomic scale by next-generation sequencing of immunoprecipitated nascent DNA. We generated one control library of input DNA and two independent biological replicate libraries of immunoprecipitated nascent DNA from EJ30 cell nuclei (termed IP-A and IP-B). These three libraries were subjected to next-generation sequencing on the Illumina HiSeq platform. We were able to map 128,887,363 sequencing reads (91.09%) to the human genome for the input control library, and 175,743,424 and 199,341,742 reads (93.68% and 93.69%) for the two immunoprecipitated nascent DNA libraries IP-A and IP-B, respectively.

A local enrichment of sequencing reads is visible over and above the corresponding input at the *MYC* gene and at the promoters of the *MCM4* and *TOP1* genes in the immunoprecipitated nascent DNA of both experimental replicates IP-A and IP-B (Figure 2B). These local enrichment sites correspond to the locations of the previously mapped broad DNA replication initiation zone at the *MYC* locus and the narrow discrete origins at the *MCM4* and *TOP1* promoters (6,7,40). Some enrichment of nascent DNA extends a few kb away from these origins in both directions, consistent with the limited movement of replication forks away from their initiation sites.

We next used the SICER algorithm (41,42) to call enrichment peaks at a genomic scale. SICER is a preferred tool for identifying enrichment peaks of the kind obtained in our immunoprecipitation experiments, because it identifies areas of enrichment in the range of a few hundred nucleotides and above, rather than calling highly focal peaks such as transcription factor binding sites. We compared two SICER modes (see Materials and Methods). The first integrates the input control DNA library into the peak-calling process whilst the second mode uses only local enrichment data in the sample library without integrating the input library. We compared the called enrichment peaks for the two independent experimental replicates IP-A and IP-B using both SICER modes, and identified the peaks found in both experimental replicates by calculating the intersection values between the two call sets (Figure 2B and C).

We first counted the numbers of called peaks at high statistical cut-off values (FDR and *E*-value both set at 1 × 10e-5), and determined the number of common peaks. More than 60,000 peaks were called for each of the two independent immunoprecipitated DNA libraries IP-A and IP-B when the input library was included in the SICER analysis, but only 54% of these peaks were present in both libraries (Figure 2C). When peaks were called without using the input library, we found about 25,000 and 50,000 peaks in the immunoprecipitated DNA libraries IP-A and IP-B, respectively. However, in contrast to the first approach, 97% of the peaks in library IP-A overlapped with those in the larger library IP-B (Figure 2C). When statistical thresholds of peak calling were relaxed, increasing numbers of peaks were identified (Supplementary Figure S3A), yet the high concordance between the biological replicates was maintained (Supplementary Figure S3B).

We next compared both modes of SICER peak calling qualitatively. The previously identified replication origins at the promoters of the *MYC, MCM4* and *TOP1* genes were reliably identified by SICER in both experimental replicates at high statistical cut-off values (Figure 2B). Any peaks found in only one replicate were successfully excluded by the intersect analysis in both SICER approaches. At the ‘laminB2 origin’, a peak was only called when excluding the input library, whereas both SICER modes identified a separate peak over the promoter of the *LMNB2* gene (Supplementary Figure S4). Taken together, we conclude that the SICER peak-calling mode that excludes the input DNA library is more sensitive with our dataset and more specific than the alternative approach that includes the input.

There are two possible explanations for this observation. First, there may have been insufficient sequencing depth for the whole-genome control input library (with 129 million reads) compared with the highly enriched immunoprecipitated libraries IP-A and IP-B (with 176 and 199 million reads). This could result in false-positive peak calling in areas of low read coverage in the input. Second, the input pre-immunoprecipitation library contains the replicated origin-proximal DNA, which might result in inefficient peak-calling due to a local increase of read density around some activated origins in the input. With these points in mind we adopted the SICER peak-calling mode excluding the control input library as the more stringent method for subsequent analyses.

**Figure 3.**
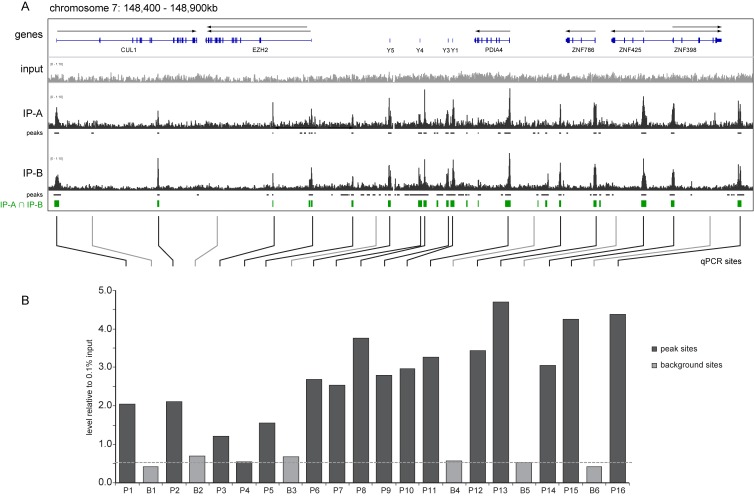
Location of DNA replication origins on chromosome 7. Analysis of origin location by (**A**) deep sequencing and (**B**) real time PCR of immunoprecipitated nascent DNA. Analysis was performed for a 0.5 Mb area on chromosome 7 (148.4–148.9 Mb), as detailed for Figure 2B. The dashed line indicates the average background signal. Sequences for the PCR primer pairs are in Supplementary Table S1 and their positions on the local genome map are marked.

As a next step we investigated a genomic region that has not previously been analysed with respect to replication origins. We selected a 0.5 Mb region on the right arm of chromosome 7 that encompasses several protein-coding genes as well as the genes encoding the highly expressed small non-coding Y RNAs (Figure 3). Areas of distinct nascent DNA enrichment over and above the background signal were observed, and we were able to identify peaks computationally over the promoters of all the protein-coding genes, at the loci of the four short Y RNA genes, and at a few additional intergenic and intronic sites (Figure 3A). To validate this analysis independently, we generated 16 PCR primer pairs to amplify sites of nascent DNA enrichment detected by both SICER approaches, as well as five non-called background sites. Quantitative PCR confirmed that 15 of 16 sites called by SICER were also amplified by qPCR over and above the level obtained at the five background sites (Figure 3B).

These results indicate that we have developed a new method for the identification of DNA replication origins at a genomic level. We call this method ‘initiation site sequencing’ (ini-seq).

### Genome-wide distribution of replication origins

**Figure 4.**
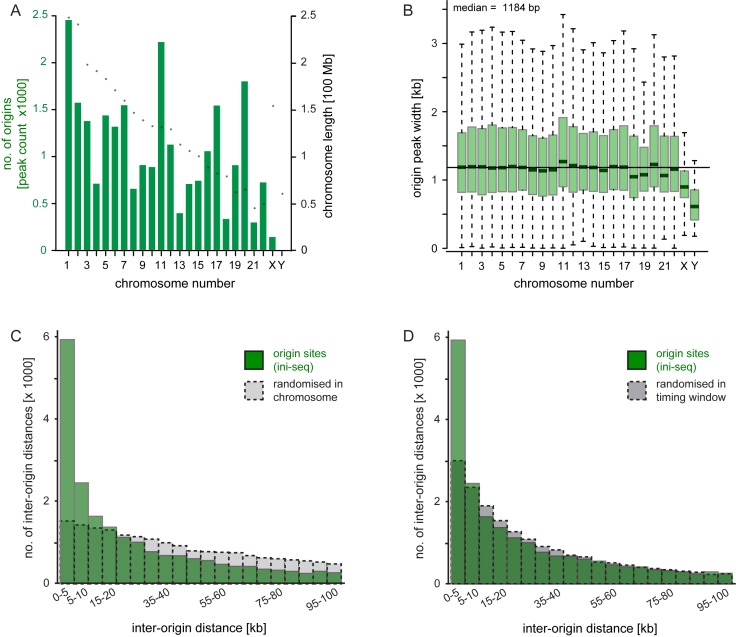
Genome-wide analysis of DNA replication origin distributions. Enrichment peaks of immunoprecipitated nascent DNA (origin peaks) were called by SICER, excluding the input DNA library. Only peaks present in both libraries IP-A and IP-B (i.e. the intersect) are subsequently analysed. (**A**) Distribution plot of origin peak counts for each individual human chromosome. Individual chromosome lengths are indicated by asterisks. (**B**) Distributions of origin peak widths for each human chromosome. Box and whisker plots are shown without individual outliers, whiskers represent 1.5 times the interquartile range above the upper quartile and below the lower quartile, and the overall genomic median is indicated. (C and D) Origin spacing. Inter-origin distances were calculated in 5 kb intervals for the distributions of activated replication origins (green) and control sites (dashed grey) that were randomised either (**C**) within each chromosome, or (**D**) within DNA replication timing windows (46).

We identified 25 054 activated replication origins in the human genome by ini-seq at the stringent statistical cutoff of *E* = 10e-5 (Figure 2C). These replication origins were distributed non-randomly across individual chromosomes and the number of origins detected per chromosome did not correlate with chromosome length (Figure 4A), as was observed in the distributions of genomic sites obtained by SNS-seq (16).

The width of an origin peak identified by ini-seq gives an indication for the extent of DNA replication from this origin. Its value would become larger when the origin fired early, when the replication forks progressed away from the initiation site at higher speeds, or when converging forks from adjacent origins fused during the replication reaction *in vitro*. The median of the peak width distribution in the intersect of the two immunoprecipitated libraries was 1184 bp, and we found no major variation of peak width distributions between the human chromosomes (Figure 4B), or between the two immunoprecipitated libraries (Supplementary Figure S5A). One exception to this observation may be the sex chromosomes, where fewer origins and shorter peak widths were observed (Figure 4A and B and Supplementary Figure S5A). We note that the EJ30 cells used as the source for template nuclei are of male origin (43) and we have therefore included the Y chromosome in our analysis despite the low mapping coverage and the low number of just 13 origins called. Overall, these data are consistent with the 15 min incubation used in our system, taking into consideration origin firing timing of 10 min and subsequent replication fork progression rates of 300 ± 200 bp/min (27,31–33).

The frequency of initiation at a particular site can be inferred from the number of reads within the called enrichment peak. The distributions of read counts for the peaks present in both libraries varied within an order of magnitude across the entire genome, and this distribution was similar for all chromosomes (Supplementary Figure S5B). Library A had half the number of sequencing reads per enrichment peak than library B, indicating that immunoprecipitation A was less efficient than B, and explaining the lower number of origins identified in library A compared with B (Figure 2C).

**Figure 5.**
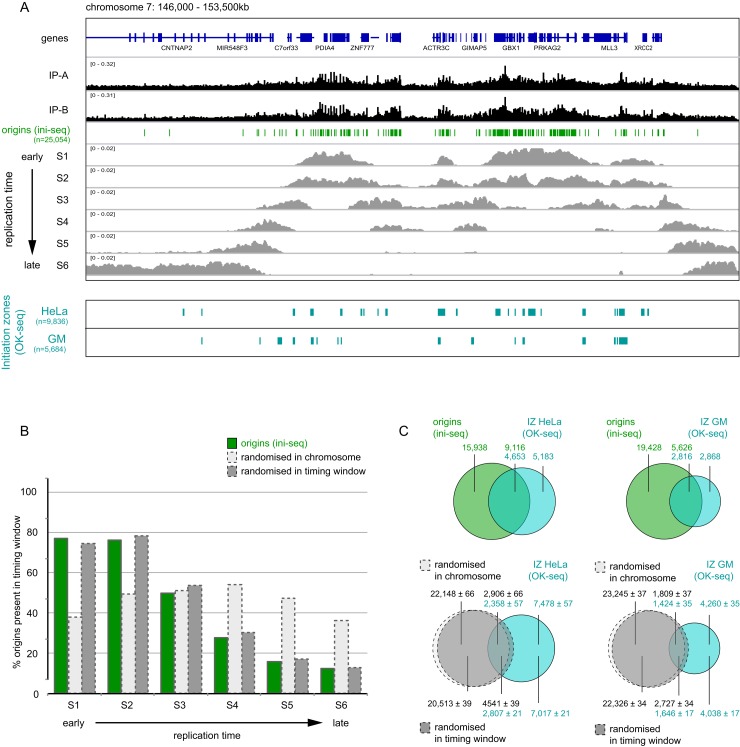
Activated origins are located predominantly in early-replicating domains of the human genome. (**A**) Positions of sequencing reads are visualised on the integrated genome viewer (IGV) for the two independent biological replicates of immunoprecipitated nascent DNA (IP-A and IP-B). Replication origins activated in vitro were called by SICER without input; green bars show peaks that are only present in both IP libraries (i.e. the intersects at *E* = 1 × 10e-5, as determined in Figure 2). The middle section shows replication timing profiles in HeLa cells taken from (46) and visualised on IGV, representing the genome replication timing windows of six consecutive stages of S phase progression, from S1 (earliest) to S6 (latest). The bottom panel shows the location of initiation zones (IZ) as determined by Okazaki fragment sequencing (OK-seq) (25). (**B**) Quantification of origin location within the six replication timing windows of S phase. The overlap was determined between each DNA replicating timing window S1–S6 and the origin sites determined by ini-seq (green), as well as control sites after randomisation within each chromosome (dashed light grey), and within DNA replication timing windows (dashed dark grey). Percentages of origin and control sites present in each timing window are plotted. Percentages for S1 to S6 add up to >100% because of overlap between individual timing windows. (**C**) Top row: Venn diagram overlap analysis of replication origins determined by ini-seq (green) with initiation zones (IZ) determined by OK-seq (light blue) in HeLa (left) and GM06990 cells (right). Bottom row: overlap analysis of control sites after randomisation within each chromosome (dashed light grey) or within DNA replication timing windows (dashed dark grey) with initiation zones (light blue). Overlays of Venn diagrams are shown for these two analyses. Absolute numbers of origins/control sites and initiation zones are indicated for each section of the Venn diagrams.

The inter-origin distances determined by ini-seq were highly heterogeneous, with a high proportion of comparatively small values (Figure 4C, green bars). To control for chance, we generated two reference distributions in which we fully randomised the start position of each origin peak either within its host chromosome (Figure 4C) or within its replication timing window (Figure 4D and see below, Figure 5). About 10 000 origins are clustered at distances shorter than predicted by chance through randomisation in the chromosome (<15 kb), with particularly short inter-origin distances of <5 kb being observed at the highest frequencies (Figure 4C and Supplementary Figure S6). Conversely, about half of the origins are spaced at intervals larger than expected by chance (>20 kb). A similar albeit less striking overrepresentation of short inter-origin distances was observed when comparing them to a control distribution in which the origin positions were randomised within their replication timing window (Figure 4D). These data suggest that activated origins tend to cluster at short intervals in discrete genomic regions. We conclude that the distances between activated origins are not distributed randomly.

### Replication timing profile of activated replication origins

Replication origins become activated throughout the 8–10 h course of S phase in human cells (3,44). The cell-free system employed in this study recapitulates the G1 to S phase transition of the cell cycle (26), so it is likely that origins activated in this system represent those that normally fire at the onset of S phase (39). To explore this question, we compared the locations of activated origins identified by ini-seq with replication timing windows of the human genome (Figure 5), determined by high-throughput sequencing of replicating DNA in six consecutive windows of S phase in HeLa cells, from early (S1) to late (S6) (45,46).

In a representative and predominantly early-replicating 7.5 Mb domain on chromosome 7, the majority of activated origins are located and clustered at high densities in the early replicating S1 and S2 domains (Figure 5A). However, active origins were also clustered in transition areas replicating in mid-S phase domains S3 and S4, and a few isolated origins were detected in late replicating domains S5 and S6 (Figure 5A). We went on to quantify the overlap between origin locations and replication times genome-wide (Figure 5B). The percentages of active origins present in a particular timing window proved to decrease from 75–80% in early S phase to 10–15% in late S phase (Figure 5B, green bars). As expected, the control origin distribution obtained after randomisation within its chromosome differed significantly, with these sites being distributed evenly across the six replication timing windows (Figure 5B, light grey bars). When compared with this randomised data set, activated origins were found to be overrepresented in early replicating and underrepresented in late replicating domains of the genome (Figure 5B).

To control for the possibility that the short inter-origin distances observed by ini-seq might be a consequence of preferential origin positions in early replicating sections of the genome, we also compared the distribution of origin positions to a control distribution in which origin positions were randomised within their timing windows. We determined the average width of an early replicating window as 480 kb (i.e. the mean of S1 and S2) and shifted the position of each origin by a randomised value between ±240 kb. This distribution maintained the replication timing profile of the original origin distribution (Figure 5B, dark grey bars), but it still showed lower proportions of the shortest inter-origin distances than the original origin distribution (Figure 4D).

We therefore conclude that activated DNA replication origins detected by ini-seq are located and cluster predominantly, but not exclusively, in early-replicating domains of the human genome.

### Activated origins overlap preferentially with early initiation zones defined by Okazaki fragment sequencing

A recent genome-wide sequencing of Okazaki fragments quantified replication fork directionality and revealed between 5000 and 10,000 broad initiation zones in two human cell lines (25). We asked whether origin sites identified at high resolution by ini-seq overlap with these initiation zones (Figure 5, and Supplementary Figures S7 and S8).

Initiation zones in the genome are defined by an upward shift of replication fork directionality from the (-) DNA strand to the (+) strand (25). Visual inspection of a representative genome region shows that several individual origins identified by ini-seq are usually present in initiation zones in HeLa and GM06990 cells (Supplementary Figure S7). Outside of initiation zones, small upward jumps or discontinuities in an otherwise downward oriented section of replication fork directionality often co-localise with isolated origins identified by ini-seq (Supplementary Figure S7), suggesting that ini-seq can also identify relatively inefficient isolated origins.

Ini-seq origins usually overlap with initiation zones in early replicating domains, but a substantial number of origins are present outside of these initiation zones (Figure 5A). At the genome-wide level, 36% and 22% of origins identified by ini-seq overlapped with initiation zones identified by OK-seq in HeLa and GM06990 cells, respectively (Figure 5C, top diagrams). Only between a third and half of this overlap is due to chance as only 12/18% and 7/11% of control sites after randomisation within chromosomes or replication timing windows overlapped with these initiation zones, respectively (Figure 5C, bottom diagrams). Conversely, about half of all initiation zones contained origins identified by ini-seq, with an average number of two origins per initiation zone (Figure 5C, top diagrams). When the analysis was restricted to initiation zones present in the early replicating domain S1 of the genome, a larger proportion of initiation zones contained origins identified by ini-seq (Supplementary Figure S8A, top row), and a lower proportion of initiation zones overlapped with control distributions after randomisation of origin positins within either chromosomes or replication timing windows (Supplementary Figure S8A, bottom row). The vast majority of origins identified by ini-seq that are present in initiation zones in GM06990 cells were also present in initiation zones in HeLa cells (Supplementary Figure S8B).

We conclude that a significant proportion of early firing origins identified by ini-seq are present in the early firing broad initiation zones identified by OK-seq. However, ini-seq analysis resulted in the identification of an additional 15–20,000 origins outside of these demarcated initiation zones determined by OK-seq.

### Activated origins overlap predominantly with transcription start sites and G4 motifs

**Figure 6.**
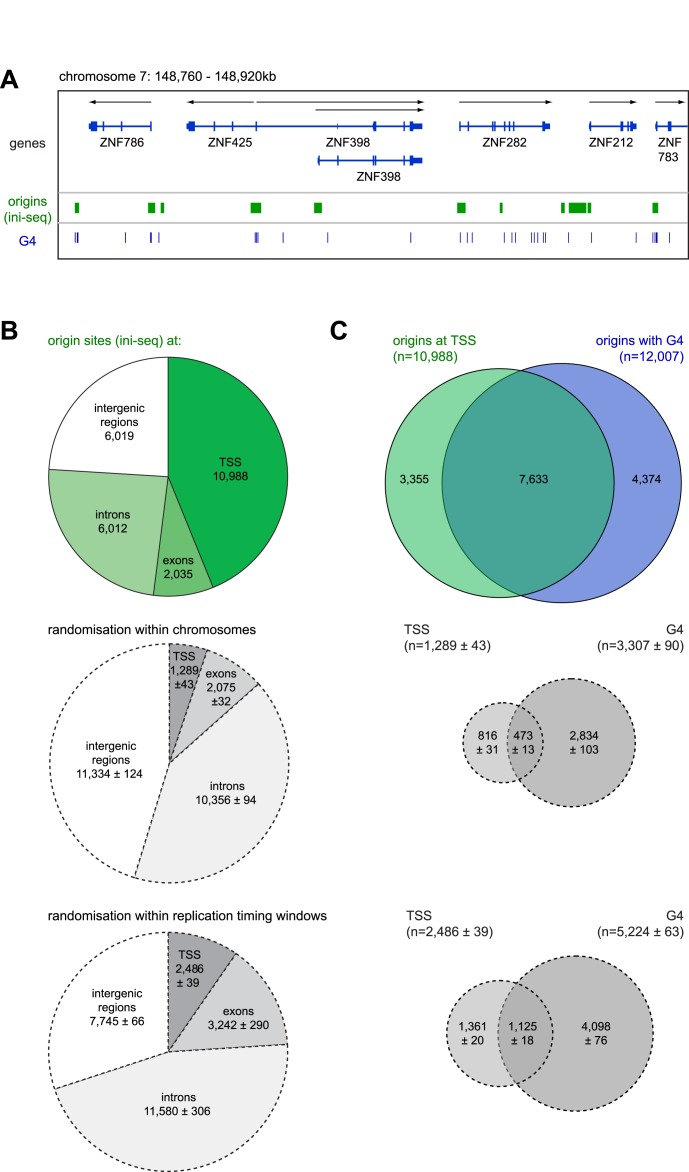
Activated origins predominantly co-localise with transcription start sites and G-quadruplex motifs. (**A**) Comparative visualisation of activated replication origin sites determined by ini-seq (green) and locations of G quadruplex motifs (G4, blue) using the integrated genome viewer (IGV). Positions of G4 motifs were obtained according to (48) and lifted over to the human genome GRChr37 release. (**B**) Overlap quantification between genetic elements and DNA replication origin sites (green pie chart; top) or control sites (dashed grey charts) after randomisation within each chromosome (middle) and within DNA replication timing windows (bottom). (**C**) Venn diagram representation of the overlap between activated replication origins present at transcription start sites (TSS, green) with origins containing G4 motifs (G4, blue). Overlap values were also determined for the corresponding control sites (dashed grey diagrams) after randomisation within each chromosome (middle) and within DNA replication timing windows (bottom).

Early replicating domains correlate with gene-dense domains of the genome (3,44), and visual inspection of several genomic regions suggests that origins determined by ini-seq frequently overlap with transcription start sites (TSS) of protein-coding and non-protein coding genes (Figures 2, 3, 5 and 6A). With this in mind we quantified the overlap between activated origins detected by ini-seq and transcriptional elements (Figure 6). At the genomic level, more than three quarters of activated origins overlapped with transcribed areas of the genome (Figure 6B, top). Almost 50% overlapped with TSS of protein-coding and non-coding genes. Within gene bodies, three times more origins were present in introns compared to exons. It might be argued that this overlap is due to chance because origins determined by ini-seq are predominantly located in early-replicating and gene-dense areas of the genome. However, this is not the case because the overlap with TSS of control sites after randomisation within each chromosome and within each replication timing window was reduced nine- and four-fold, respectively (Figure 6B, mid and bottom). In contrast, the overlap of these control sites with intergenic regions and introns increased accordingly. These data indicate that activated origins determined by ini-seq are strongly and specifically enriched at TSS.

Single stranded DNA or RNA can form a G quadruplex (G4) when four blocks of three or more consecutive guanosines, separated by variable loop lengths, form a stack of guanosine tetrads (48). Genome-wide SNS-seq analyses have correlated the presence of these G4 motifs with human replication origins (16,17,22), and genetic manipulations of two candidate loci in chicken cells provided functional evidence that G4 motifs are necessary regulatory elements for origin function (47). We therefore asked whether G4 motifs are also found at origins identified by ini-seq (Figure 6A and C). We identified 359,267 G4 motifs in the human genome and compared them with the 25,054 origin positions determined by ini-seq. Strikingly, the large majority of origins at TSS also contained G4 motifs, and of the origins containing G4 motifs, the majority were present at TSS (Figure 6C, top). This overlap is not due to chance because the much smaller numbers of sites in the control distributions after randomisation within chromosomes or replication timing windows, that were present at TSS or contained G4 motifs, failed to show substantial overlap (Figure 6C, mid and bottom). We conclude that a large proportion of activated origins determined by ini-seq are not only present at TSS but also specifically contain G4 motifs.

### Overlap between DNA replication origin locations determined by ini-seq, SNS-seq and bubble-seq

**Figure 7.**
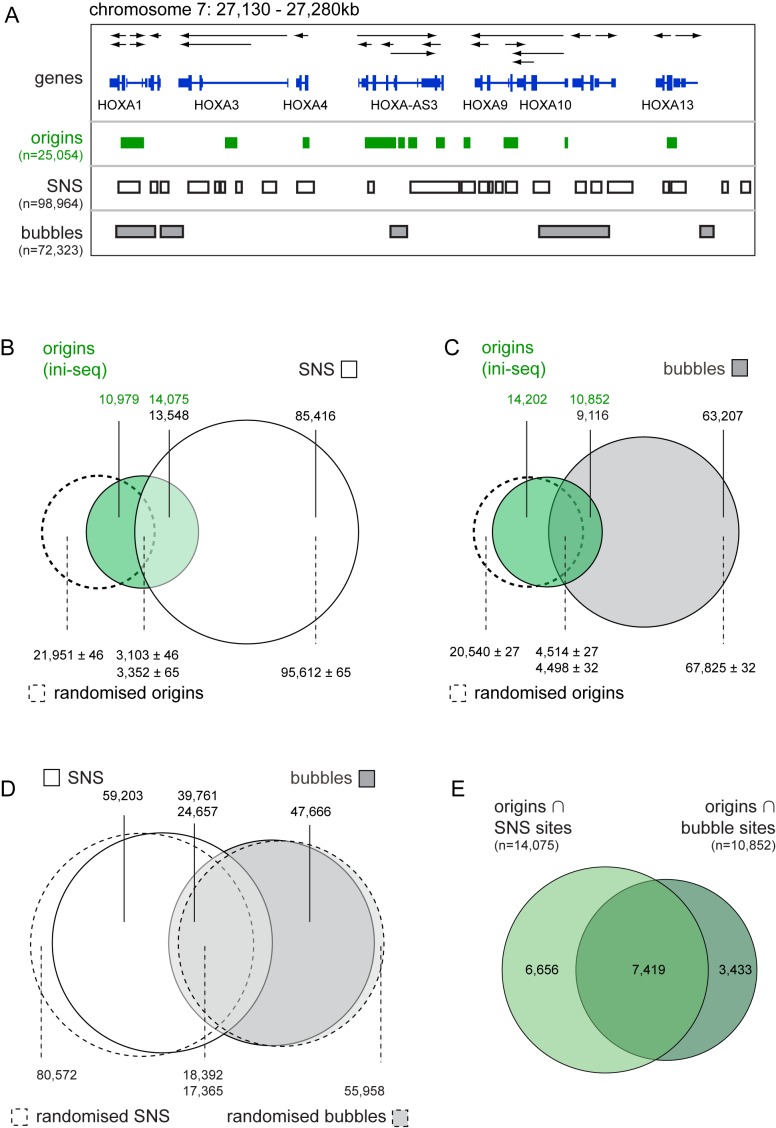
Overlap between DNA replication origins and genomic sites determined by SNS-seq and bubble-seq. Original SNS-seq (16) and bubble-seq (23) data were lifted over to human genome release GRChr37. Genomic positions of SNS-sites were determined by SICER peak calling from the original sequencing data of (16), and genomic positions of restriction fragments containing replication bubbles were generated by combining results from three replicates of (23). (**A**) Comparative visualisation of activated replication origins identified by ini-seq (green), SNS sites (boxed white) and bubble fragments (boxed grey) at the HOX gene locus on the integrated genome viewer (IGV). (**B** and **C**) Venn diagram overlap analysis between origins identified by ini-seq (green) and sites determined by SNS-seq (B, white) and bubble-seq (C, grey). Overlap analyses of control sites derived from ini-seq analysis after randomisation within each chromosome (dashed) with SNS-seq and bubble-seq sites are superimposed. (**D**) Overlap between genomic positions of SNS sites and bubble fragments. Overlap analyses of control sites derived from SNS-seq and bubble-seq analyses after randomisation within each chromosome are superimposed (dashed). (**E**) Overlap between the intersect of origins identified by ini-seq and SNS sites (brighter green) and the intersect of origins and bubble-seq fragments (darker green). Numbers of detected sites are indicated for each section of the overlap analyses.

SNS-seq and bubble-seq analyses of replication origins show rather limited concordance (3), so we went on to compare these approaches with our ini-seq data (Figure 7). To allow direct comparison between the three methods of analysis, we lifted over the genomic coordinates from the original human HeLa cell SNS-seq data (16), and GM06990 cell bubble-Seq data (23) to the same genome release used for our data (GRCh37).

We first analysed the original genomic SNS-sequencing data (16) by SICER, using the same stringency definition previously applied to our ini-seq data. We obtained 98,964 peaks, fewer than the number reported originally (16), but more than were identified by two recent re-analyses of the same original data set involving a clustering approach (17,19). Examination of the *HOX* gene locus revealed good visual concordance between sites identified by SICER (Figure 7A) and those identified by the original Sole-Search peak calling approach (shown in Figure 1A of the original publication (16)).

At the genome-wide level, more than half of the origins identified by ini-seq (56%) overlapped with sites identified by SNS-seq, but only 14% of the much larger number of SNS-seq peaks overlapped with ini-seq origins (Figure 7B). Similar percentages of reciprocal overlap (51–57% and 12–14%, respectively) were seen between ini-seq sites and SNS-seq sites determined in three different human human cell types (16) (IMR-90, induced pluripotent stem cells from IMR-90, and human embryonic stem cells H9), suggesting that different cell types contribute only limited variability to the outcome of this analysis. The overlap between ini-seq and SNS-seq sites is not significantly due to chance because just 12% of sites in the randomised control distribution overlapped with the SNS sites (Figure 7B).

To compare our ini-seq data with the genomic sites identified by bubble-seq, we first combined results from the three replicates in the original data (23) into a single data set containing 72,323 unique bubble-fragments. We observed moderate concordance between these sites and our ini-seq data: only 43% of the origins identified by ini-seq overlapped with sites identified by bubble-seq, and 13% of bubble-seq fragments overlapped with ini-seq origins (Figure 7C). Furthermore, about half of the overlap between ini-seq and bubble-seq sites is due to chance, because 18% of sites in the randomised control distribution also overlapped with restriction fragments containing replication bubbles (Figure 7C). In comparison, 40% of SNS-seq peaks and 35% of bubble fragments overlapped with each other, and more than half of this overlap was due to chance (Figure 7D). Finally, we found that the majority of ini-seq origins overlapping with SNS sites also overlapped with bubble-fragments (Figure 7E). Of these 7419 origin sites identified by all three approaches, 57% also overlapped with TSS and 70% with G4 motifs. Interestingly, these common sites overlapped with initiation zones determined by OK-seq in HeLa and GM06990 cells to similar proportions (42% and 27%) than all ini-seq origins did (36% and 22%, see Figure 5C), arguing that OK-seq analysis does not specifically select further for these common sites.

We conclude that origin sites identified by ini-seq have the highest and most specific concordance with sites identified by SNS-seq, followed by those identified by OK-seq and bubble-seq. Significantly, ini-, SNS- and bubble-seq identify a subset of replication origins that are located at transcription start sites and enriched in G4 motifs.

### Overlap of nucleotide compositional skew jumps with DNA replication origins

**Figure 8.**
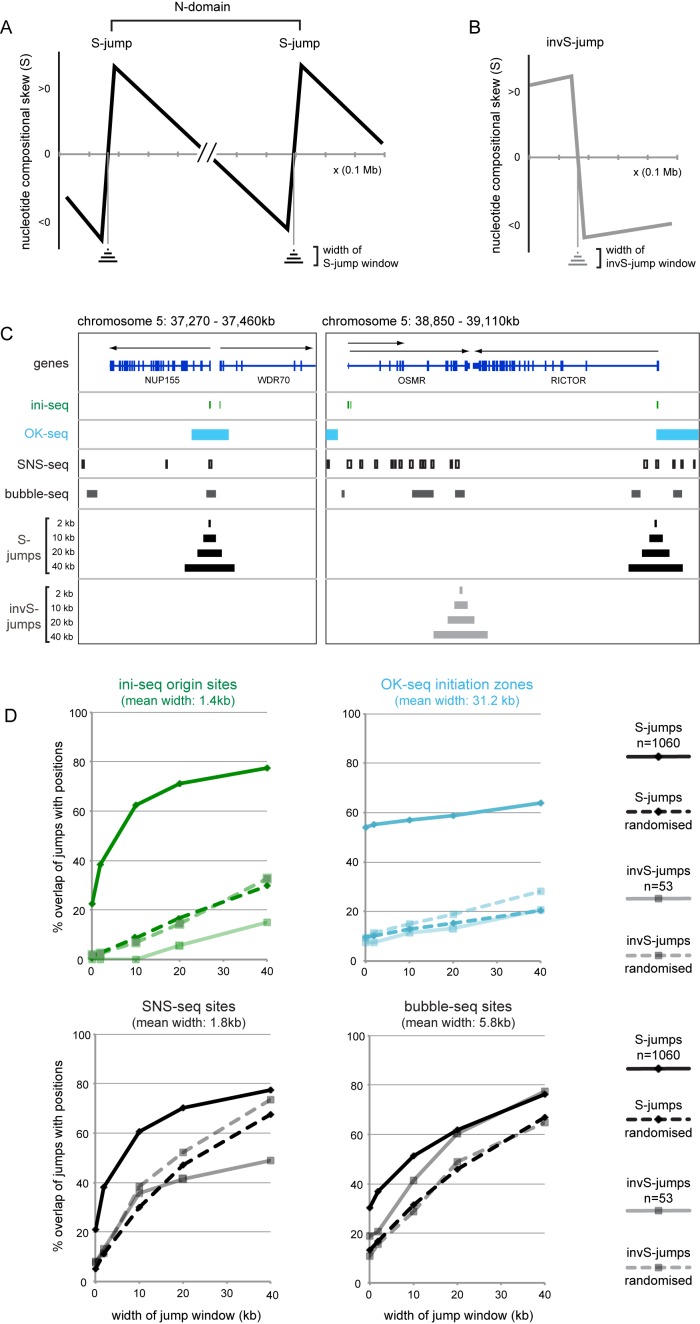
Overlap of nucleotide compositional skew jumps with DNA replication origins. (**A**) Schematic representation of nucleotide compositional skew at two consecutive positive S-jumps demarcating an N-domain in the genome, adapted from (34,35). The nucleotide compositional skew is defined as: S = (T-A)/(T+A)+(G-C)/(G+C). Different misincorporation rates of nucleotides on the leading and lagging strand in the germline eventually result in a positive skew shift (S-jump) at the position of a localised replication origin. S-jumps span up to several tens of kilobases, so a nested set of size windows up to 40kb was generated around the nominal jump nucleotide and used for overlap determinations to facilitate comparisons with distributions origin sites of different mean widths. (**B**) Schematic representation of an inverted S-jump arising from a localised replication terminator element or from transcription end points in the absence of a replication origin, adapted from (34,35). (**C**) Comparative visualisation of skew jumps with activated origins determined by ini-seq (green), initiation zones determined by OK-seq (light blue), SNS-seq sites (boxed white) and bubble-seq sites (dark grey). Positions of positive S-jumps (black, *n* = 1060) and inverted S-jumps (light grey, *n* = 53) were obtained as the terminal nucleotide positions of N-domains and inverted N-domains (34), and lifted over to the human genome GRChr37 release. Nested jump size windows around the skew jumps (in kb) are indicated. (**D**) Overlaps of positive S-jumps (dark hues) and inverted negative S-jumps (light hues) with sites determined by ini-seq (top left panel), OK-seq (top right), SNS-seq (bottom left) and bubble-seq (bottom right). Solid curves show the overlap of S-jumps and inverted S-jumps with the original ini-, OK-, SNS- and bubble-seq sites, while dashed curves with the randomised distributions of these sites. The average widths of these sites are indicated for comparison.

We finally compared the sites of activated replication origins identified by ini-seq with locations of potential replication origins predicted by an entirely computational approach (Figure 8). The nucleotide composition of the genome is skewed, and a systematic determination of the nucleotide compositional skew, defined as S = (T-A)/(T+A)+(G-C)/(G+C), has identified 663 discrete domains in the human genome (35,49). Averaged over 1 kb windows, these domains are flanked by sudden positive increases of the skew, with gradual negative declines between them. Because of their shape, these are called N-domains (Figure 8A). The flanking positive S-jumps are predicted to be the result of highly localised replication origins that have been active over an evolutionary time-scale in the germline, because different nucleotide misincorporation rates exist for the leading and the lagging strands of the two diverging replication forks (34,35,50). The amplitude of S-jumps is increased by a transcriptional contribution when replication origins overlap with promoters of transcriptionally active genes in the germline (34). A much smaller number of inverted N-domains has also been described that are flanked by inverted negative S-jumps (Figure 8B). Inverted negative S-jumps can arise at localised replication termination sites or transcription termination sites of converging genes (34).

We derived the genomic locations of 1060 positive S-jumps and 53 inverted S-jumps after lifting over the original coordinates (34) to genome release GRCh37, and then compared their locations with the replication origin sites identified by ini-seq, OK-seq, SNS-seq and bubble-seq (Figure 8C and D). Because S-jumps are determined as averages of 1 kb windows and span several kilobases (34,35), we generated for the purpose of this overlap analysis a set of nested windows of increasing size up to 40 kb, each centred on the actual S-jump (Figure 8). This facilitates comparison with distributions of origin sites of different average widths.

At representative genome locations there was good visual concordance of S-jumps with origins identified by ini-seq, OK-seq, SNS-seq and, to a lesser extent, bubble-seq, as well as with TSS (Figure 8C). In contrast, inverted S-jumps did not appreciatively overlap with ini-seq origins or initiation zones identified by OK-seq, but did frequently overlap with signals obtained by SNS-seq and bubble-seq, as well as with transcription termination sites (Figure 8C). We quantified the overlaps for all available positive and inverted S-jumps with the sites identified by the four origin-mapping techniques (Figure 8D, solid lines). To control for chance overlap, we also generated randomised distributions for the datasets of the four origin-mapping techniques and quantified the overlap of positive and inverted S-jumps with these randomised distributions (Figure 8D, dashed lines). We found specific overlap of S-jumps with ini-seq origins and with OK-seq initiation zones across all window sizes. The percentages of these overlaps were 3- to 5-fold higher than the overlaps with the randomised distribution (Figure 8D, top panels). S-jumps overlapped at similar proportions with SNS and bubble-seq sites, but the overlap was only slightly above the overlap with the corresponding randomised distributions, arguing that these overlaps are less specific than the overlap with ini-seq sites (Figure 8D, bottom panels).

Inverted S jumps showed no overlap with ini-seq origins within a 10 kb window, and only limited overlap at larger window sizes (Figure 8D, top left). Strikingly, this limited overlap was more than 2-fold below the overlap with the randomised control, suggesting that activated origins are excluded from inverted S-jump sites. A similar situation was observed for the initiation zones determined by OK-seq (Figure 8D, top right). In contrast, inverted S-jumps showed intermediate and even high overlap with SNS-seq and bubble-seq sites, respectively (Figure 8D, bottom panels). Inverted S-jumps overlapped with the corresponding randomised distributions to the same extent at small window sizes, and then either fell or actually rose above the randomised control at large window sizes for SNS and bubble-seq sites, respectively (Figure 8D).

Taken together, we conclude that ini-seq provides a powerful and high-resolution experimental validation for the origin sites predicted by positive S-jumps. These sites also show a similarly specific overlap with initiation zones determined by OK-seq, as reported by Petryk and colleagues (25), but at twenty-fold lower resolution due to the larger average widths of these initiation zones compared to the ini-seq origins. Finally, SNS-seq and bubble-seq sites also overlap with S-jumps, but at much reduced specificity, and both have additional and substantial contributions from other genomic sites including replication and transcription termination sites that are marked by inverted S-jumps.

## DISCUSSION

In this paper, we have described the novel approach of DNA replication initiation site sequencing (ini-seq) to map human DNA replication origins on a genome-wide basis. It is built on the direct labelling and subsequent immunoprecipitation of newly replicated DNA, synthesised a few minutes after highly synchronous initiation in a cell-free system. Depending on computational stringency criteria, we have identified >25,000 distinct sites where DNA replication forks become established on the human genome. The sites cluster in gene-rich areas in early-replicating domains. Most are found at transcriptional start sites (TSS) and are enriched for the presence of G-quadruplex (G4) motifs. Our genomic map of activated DNA replication origins complements other datasets based on sequencing isolated small nascent leading strands (SNS-seq) (16–19), Okazaki fragments (OK-seq) (25) and agarose gel-trapped replication bubbles (bubble-seq) (23). Furthermore, activated origins identified by ini-seq prove to overlap specifically with nucleotide distribution skew jumps in the genome (34), providing experimental evidence for the computational prediction that these sites constitute discrete DNA replication origins that are active over evolutionary timescales.

### Origin activation

The number of activated origins detected by ini-seq is in the range of the number of replicons per cell determined by early fibre autoradiography experiments (1,2). Significantly, ini-seq data sets are derived from a population of a few million nuclei initiating DNA replication as a cohort. Comparisons of cohort experiments with single molecule DNA fibre analyses suggest that in a cohort of cells not every potential origin is activated in every nucleus in every S phase (3,51). Thus not all of the origins detected by ini-seq will be activated in every nucleus within the cohort, consistent with observed heterogeneities of peak heights, qPCR enrichments and integrated read counts per origin peak. We therefore conclude that ini-seq provides a map of origins that are activated in the population of template nuclei used in the cell-free system, but not every site will necessarily be used as an origin in each nucleus.

Ini-seq uses template nuclei from human cells that are reversibly synchronised in late G1 phase by mimosine (27,29). About 50% of nuclei in this preparation are true G1 phase nuclei that initiate DNA replication in an origin-specific manner *in vitro*, whilst <5% of them are contaminating early, mid and late S phase nuclei (27,31–33). These S phase contaminants contribute to a dispersed background noise of nascent DNA that is synthesised by already elongating, or by *de novo* initiated replication forks. Together with the contaminating unlabelled DNA, this heterogeneous background material therefore precludes a unanimous interpretation of the background signal as evidence for dispersed initiation events between discrete origins in our system. However, we cannot rule out that dispersive initiation events may occur to some extent in addition to site-specific initiation events at activated origins. In any case, ini-seq does provide clear evidence for site-specific initiation events in human cell nuclei.

Activated origins are located predominantly in early replicating areas of the genome, they show very good concordance with early initiation zones identified by OK-seq (25), and a significant proportion of origins overlap specifically with positive S-jumps, which are present in early replicating timing domains (52,53). We conclude that ini-seq is a powerful technique to identify early firing origins, but origins firing late in S phase will be underrepresented in ini-seq data sets. However, ini-seq analysis does detect some active origins in mid- and late replication areas, suggesting that certain late firing origins can also fire early under our experimental conditions. In addition, it remains a possibility that some minor peaks in mid- and late replicating areas could be due to efficient initiation events in the few contaminating S phase nuclei (33). Finally, we cannot rule out that origins that fire rarely in early S phase in untreated, unsynchronised cells may become overrepresented in our dataset because of the synchronisation procedure required for ini-seq analysis (27,29).

We have found several clusters of highly localised discrete individual origins that become activated at distances of a few kb of each other in gene-rich domains that replicate at the same time in S phase. Close proximity of early-firing origins has also been described recently for SNS-seq data sets derived from human and rodent cells (16,17). The activation of individual discrete origins within these dense origin clusters would be detected as a broader ‘initiation zone’ when using lower resolution techniques, including 2D gels, replication timing analysis, determination of replication fork polarity by OK-seq or the sequencing of trapped replication bubbles (3,23,25). This pattern of several discrete initiation sites present within a larger ‘initiation zone’ is reminiscent of early observations of DNA replication initiation events in the DHFR gene locus in Chinese hamster cells, or in the non-transcribed spacer of vertebrate rRNA genes (54–56). A similar situation is seen by ini-seq analysis at the *MYC* locus at smaller scale, where the entire initiation zone has been called as one origin, yet closer inspection highlights discrete narrow enrichment areas as preferred initiation sites within the zone (Figure 2), confirming earlier observations (40).

Our data thus support a model in which clustering of individual replication origins into initiation zones correlates with, and perhaps causes, early replication of the associated chromatin domain. Our data do not allow us to determine how many individual origins within an initiation zone fire in the same individual nucleus, but computational analyses of OK-seq datasets indicate that broad initiation zones typically support a single but randomly located initiation event (25). Our data, together with SNS-seq data (16), argue for a clustering of discrete individual initiation sites within these initiation zones. It is therefore likely that clusters of individual and discrete origins with a low probability of firing contribute to the broad initiation zone profiles detected by OK-seq (25).

### Comparison with SNS-seq and bubble-seq

We observe good concordance of genomic sites identified by ini-seq and SNS-seq (16). The two techniques revealed comparable ranks of origin density distributions per individual chromosome, and comparable preferences for high origin densities in early replicating domains, and both resulted in the identification of about 10 000 origins overlapping with TSS within a 1 kb window. However, SNS-seq identified more discrete sites than ini-seq, even when the same computational peak calling method was applied to both datasets. On the one hand, this difference could be explained by the comparatively more efficient identification of late firing origins by SNS-seq, or by the technical size selection step used to enrich for short nascent strands in the SNS-seq protocol (16,17,19). On the other hand, however, SNS-seq may also generate additional false positive sites as result of inefficient digestion by lambda exonuclease of GC-rich sequences on DNA single strands (57). Origin calling by ini-seq is not subject to this reservation because it does not employ lambda exonuclease digestion of isolated nascent DNA.

Origins identified by ini-seq show lower concordance with genomic sites identified by OK-seq (25) and bubble-seq (23) than with those identified by SNS-seq, with decreasing specificity and increasing contributions from chance overlap with a randomised distribution. This decreasing specificity might be explained in part by the larger widths of OK-seq and bubble-seq sites compared with the narrow sites obtained by ini-seq and SNS-seq. The lower concordance might also be explained by the preference of ini-seq for detecting early firing origins, while OK-seq (25) and bubble-seq also detect late firing origins (23). Furthermore, bubble-seq analysis is biased against detecting origins on small restriction fragments and against origins located asymmetrically on these fragments (23). Such origins would be identified by ini-seq, but missed by bubble-seq. On the other hand, S-jump analysis suggests that bubble-seq also identifies genomic sites other than replication origins, such as termination or random sites. Likewise, only a very small difference was noted between bubble-seq results and a mean random values model (23), suggesting that the bubble-seq method is prone to false-positive origin identification. However, it is conceivable that bubble-seq detects origins in somatic cells that are inefficient or inactive in the germline, and that would therefore not result in the formation of S-jumps over evolutionary time-scale.

### Active replication origins overlap with TSS and G4 motifs

Ini-seq, SNS-seq, bubble-seq and OK-seq all detect a subset of discrete genomic origin sites close to TSSs. This colocation on the genome of DNA replication and transcription initiation sites is also found in many DNA tumour viruses such as SV40 and HPV (58). This architectural principle provides for co-linearity of the progression of DNA replication forks and transcription complexes, and may have evolved to prevent their head-on collision and potential collapse (44).

Of about 11 000 origins detected by ini-seq at TSSs, 7600 also contained G4 motifs, a figure that agrees with results obtained by SNS-seq (16,17). Importantly, ini-seq is not subject to potential bias of GC-enrichment by lambda exonuclease treatment (57). Our findings therefore provide independent validation of the SNS-data based observations of origin G-rich element (OGRE) and G4 motif enrichment near replication origins (16,17,22,59).

The functional significance of G4 motifs for origin function, however, is controversial. G4 motifs were shown to be essential, but not sufficient, for the activation of two replication origins in chicken DT40 cells (47). The orientation of the G4 motifs determined the position of the DNA replication start site in this system. This study furthermore showed that in addition to the G4 motifs a second 200 bp *cis*-regulatory DNA sequence element is also required for origin function (47). However, a recent study of allele-specific origin activation concluded that initiation efficiency does not correlate with the presence of G4 motifs (60). A small subset of origins in which G4 motifs were disrupted by SNPs or indels on one allele were not significantly less active than those origins on the corresponding allele containing G4 motifs (60). A functional analysis of genome-wide origin activation using compounds that stabilise or destabilise G4 structures would help resolve this controversial topic.

### Ini-seq allows functional genome-wide analyses

The biochemically controlled cell-free approach of ini-seq offers the unique advantage of allowing functional genome-wide studies of origin activation. Fractionation/reconstitution and depletion experiments (31,61,62) allow for selective removal and/or supplementation of candidate factors and study their effects on origin site selection and origin activation. The lack of any requirement for membrane permeability or cell viability (27) allows investigations of small molecule or antibody-based compounds on origin activation, for instance those that affect G4 structure formation. These studies are only partially possible or not possible at all in previously used whole cell-based systems (16,17,19,23,25). Ini-seq therefore has the potential to transform genome-wide investigations of origin mapping from a descriptive and correlative to a functional level.

## Supplementary Material

SUPPLEMENTARY DATA
