# Supplementary material for: Genome-wide identification and characterisation of human DNA replication origins by initiation site sequencing (ini-seq)
Source: Nucleic Acids Res. 2016 Sep 1;44(21):10230–47. doi: 10.1093/nar/gkw760 (PMC5137433; doi:10.1093/nar/gkw760)
Supplement: SUPPLEMENTARY DATA [file supp_44_21_10230__index.html]

Genome-wide identification and characterisation of human DNA replication origins by initiation site sequencing (ini-seq) — SUPPLEMENTARY DATA 

# Genome-wide identification and characterisation of human DNA replication origins by initiation site sequencing (ini-seq)

## SUPPLEMENTARY DATA

- SUPPLEMENTARY DATA
- SUPPLEMENTARY DATA
- SUPPLEMENTARY DATA
