# Supplementary material for: Genome-wide identification and characterisation of human DNA replication origins by initiation site sequencing (ini-seq)
Source: Nucleic Acids Res. 2016 Sep 1;44(21):10230–47. doi: 10.1093/nar/gkw760 (PMC5137433; doi:10.1093/nar/gkw760)
Supplement: SUPPLEMENTARY DATA [file supp_gkw760_nar-01731-v-2016-File010.pdf]

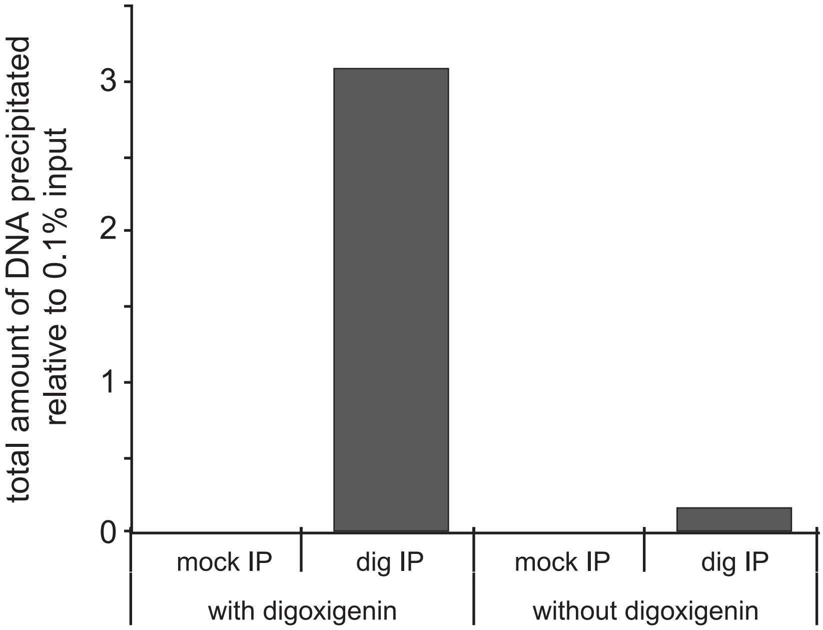
total amount of DNA precipitated relative to 0.1% input
3
2
1
0
mock IP
dig IP
mock IP
dig IP
with digoxigenin
without digoxigenin

Figure S1

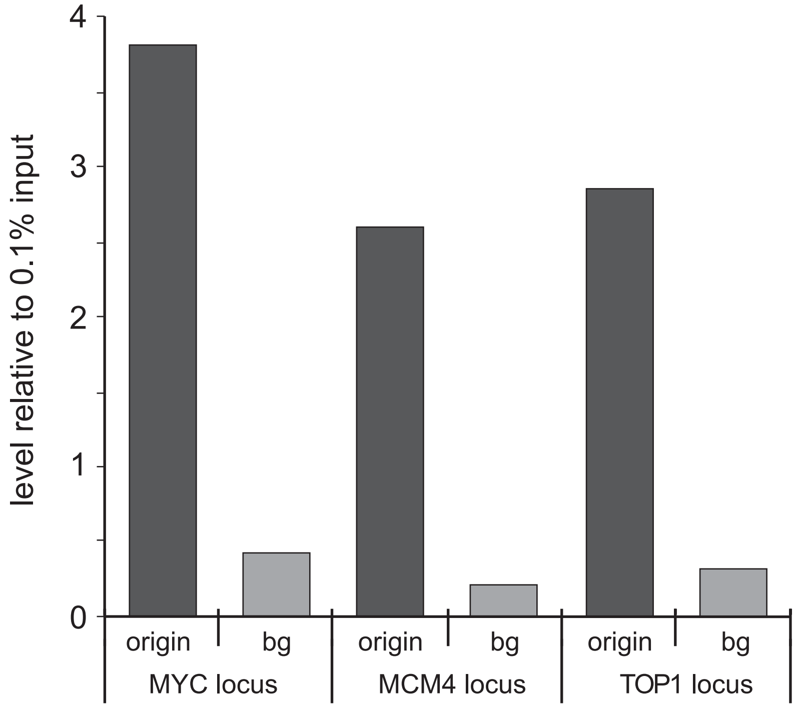
level relative to 0.1% input
4
3
2
1
0
origin
bg
origin
bg
origin
bg
MYC locus
MCM4 locus
TOP1 locus

Figure S2

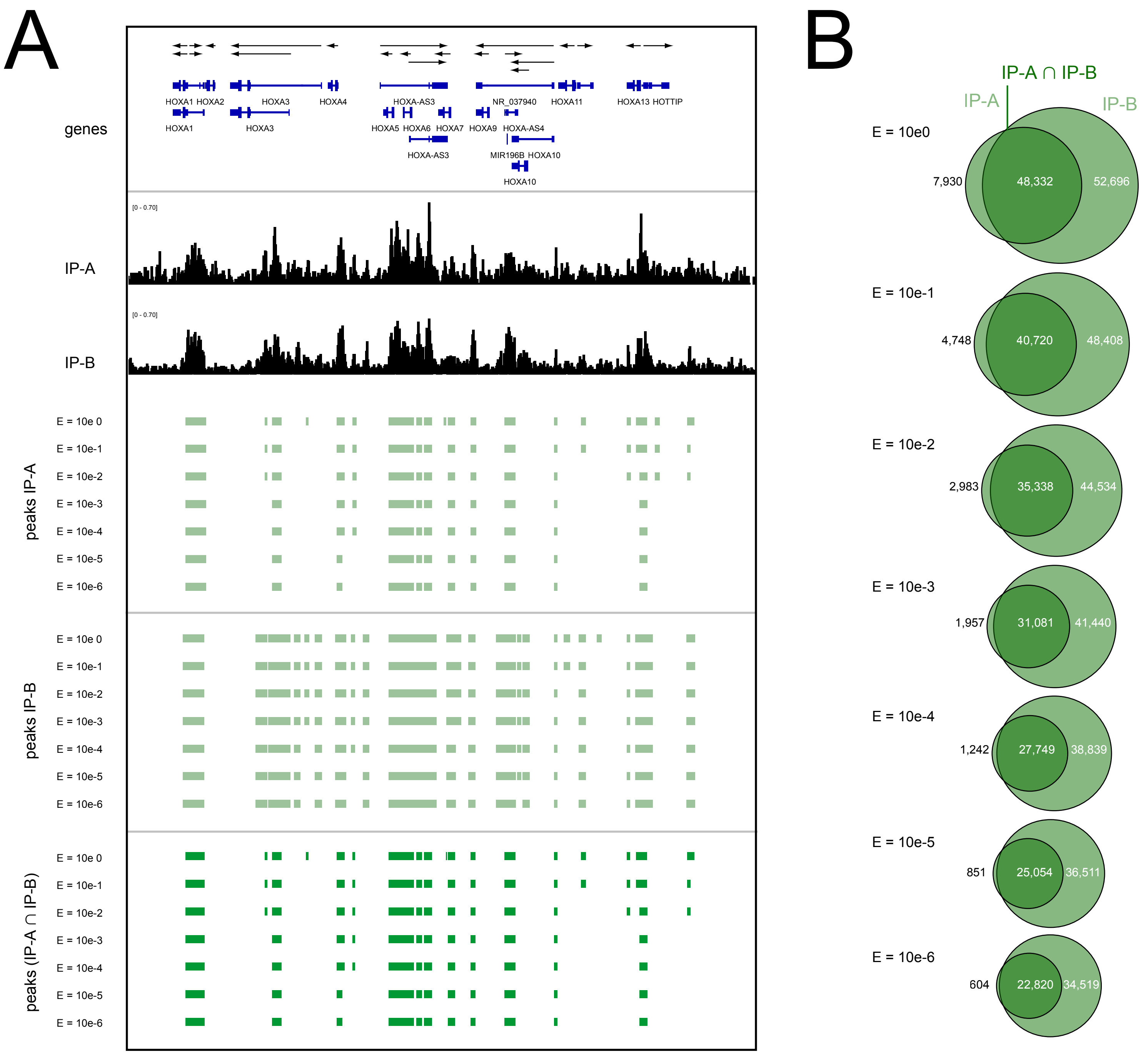
A
genes
HOXA1 HOXA2
HOXA3
HOXA4
HOXA1
HOXA3
HOXA-AS3
HOXA5 HOXA6 HOXA7
HOXA-AS3
NR_037940
HOXA11
HOXA9 HOXA-AS4
MIR196B HOXA10
HOXA10
HOXA13 HOTTIP
IP-A
IP-B
[0 - 0.70]
peaks IP-A
peaks IP-B
peaks (IP-A ∩ IP-B)
E = 10e 0
E = 10e-1
E = 10e-2
E = 10e-3
E = 10e-4
E = 10e-5
E = 10e-6
B
IP-A ∩ IP-B
IP-A
IP-B
E = 10e0
7,930
48,332
52,696
E = 10e-1
4,748
40,720
48,408
E = 10e-2
2,983
35,338
44,534
E = 10e-3
1,957
31,081
41,440
E = 10e-4
1,242
27,749
38,839
E = 10e-5
851
25,054
36,511
E = 10e-6
604
22,820
34,519

Figure S3

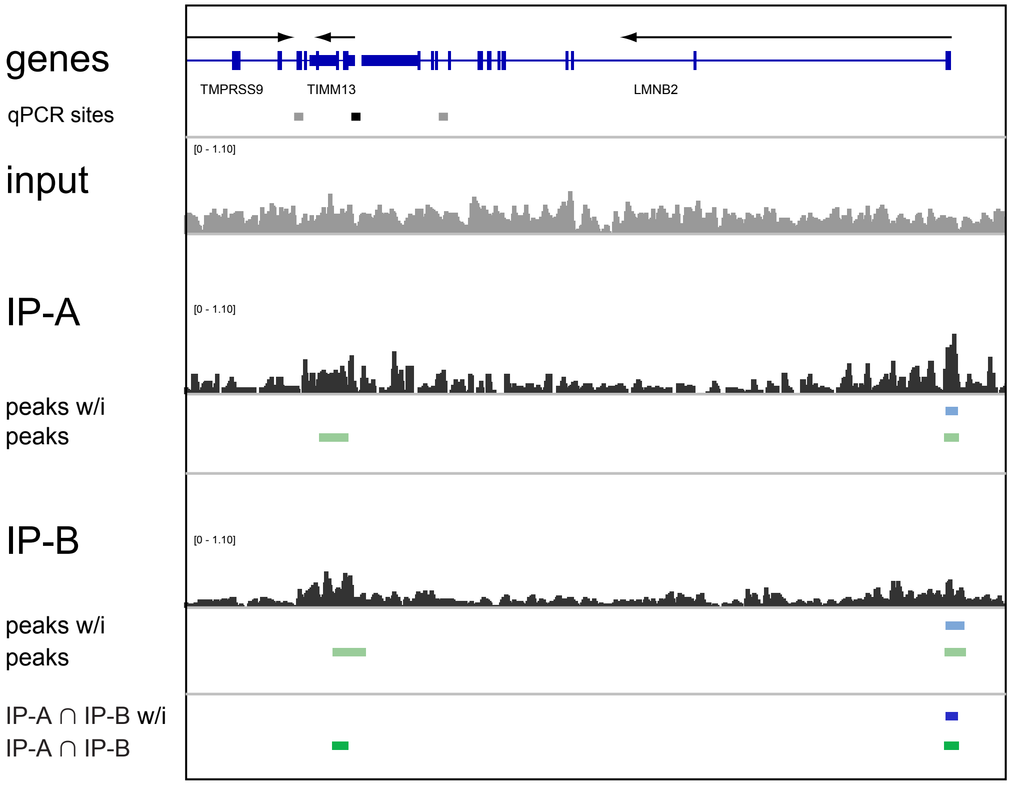
genes
TMPRSS9
TIMM13
LMNB2
qPCR sites
input
[0 - 1.10]
IP-A
[0 - 1.10]
peaks w/i
peaks
IP-B
[0 - 1.10]
peaks w/i
peaks
IP-A ∩ IP-B w/i
IP-A ∩ IP-B

Figure S4

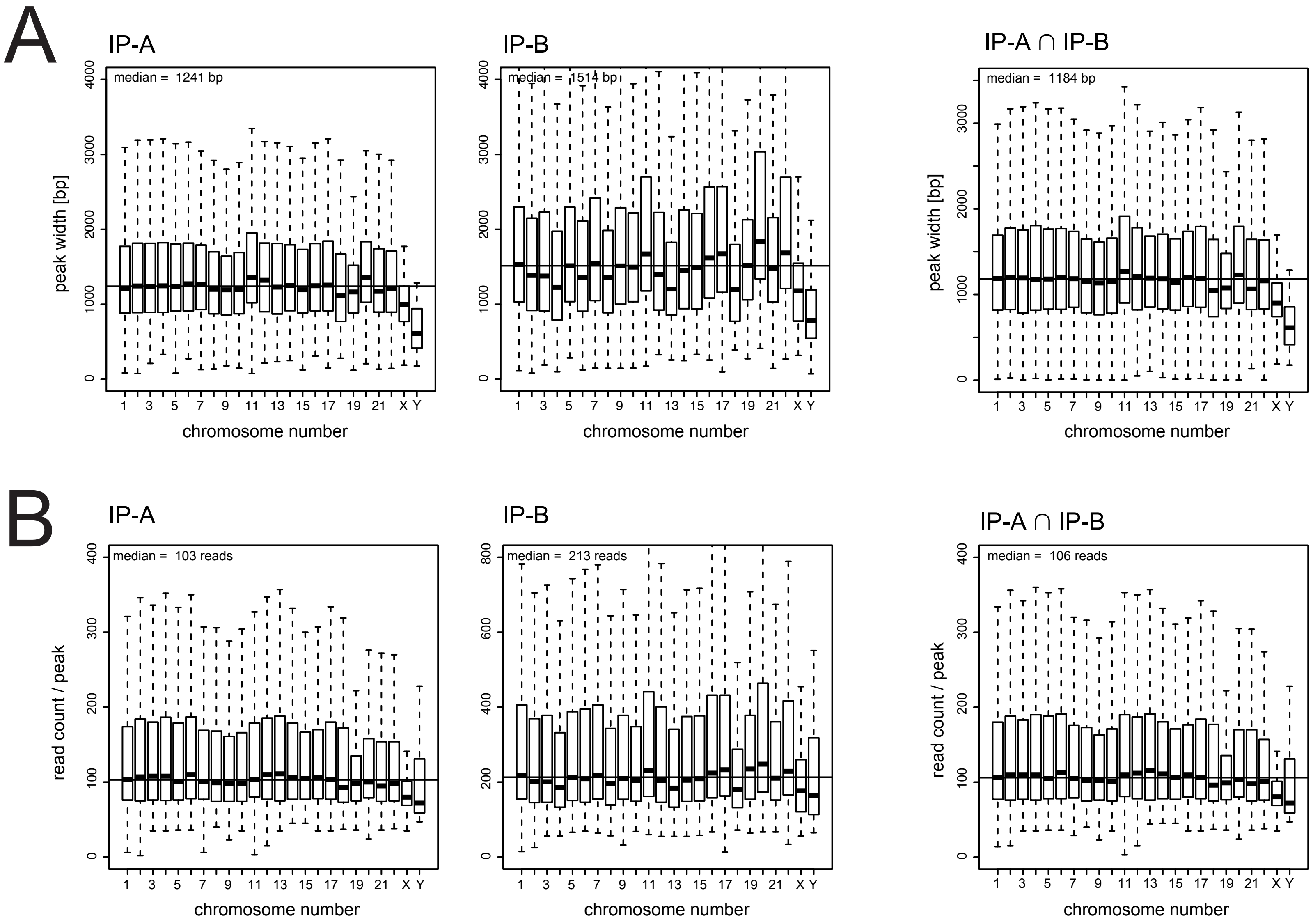
A
IP-A
median = 1241 bp
peak width [bp]
chromosome number
IP-B
median = 1514 bp
chromosome number
IP-A ∩ IP-B
median = 1184 bp
peak width [bp]
chromosome number
B
IP-A
median = 103 reads
read count / peak
chromosome number
IP-B
median = 213 reads
chromosome number
IP-A ∩ IP-B
median = 106 reads
read count / peak
chromosome number

Figure S5

A

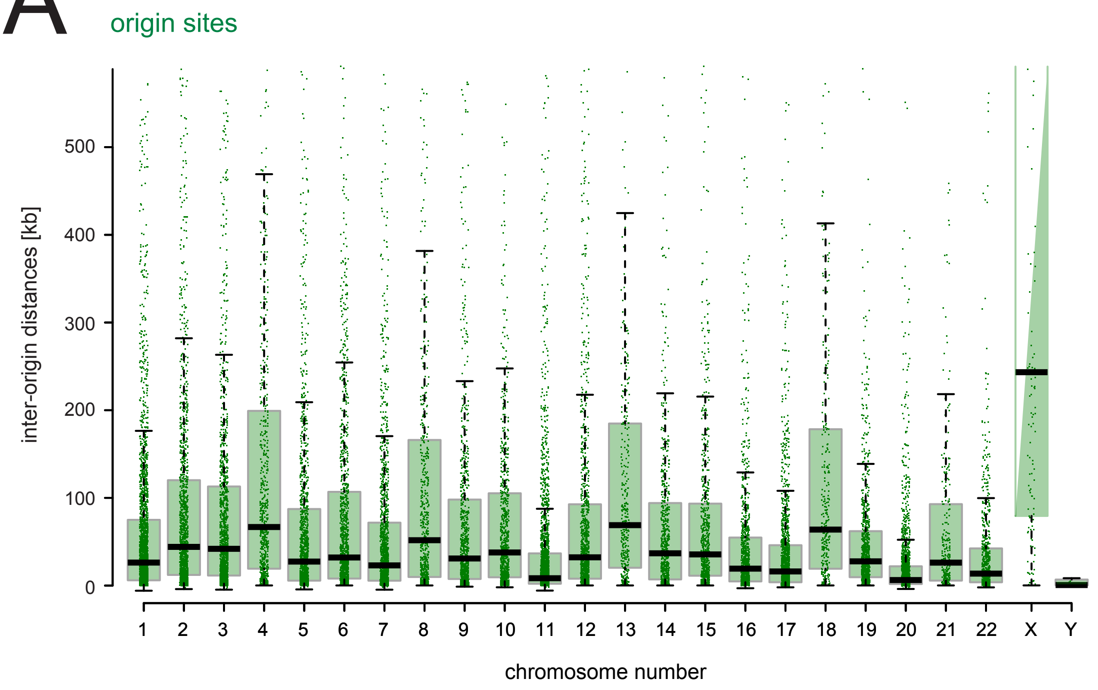
origin sites
inter-origin distances [kb]
0
100
200
300
400
500
1 2 3 4 5 6 7 8 9 10 11 12 13 14 15 16 17 18 19 20 21 22 X Y
chromosome number

B

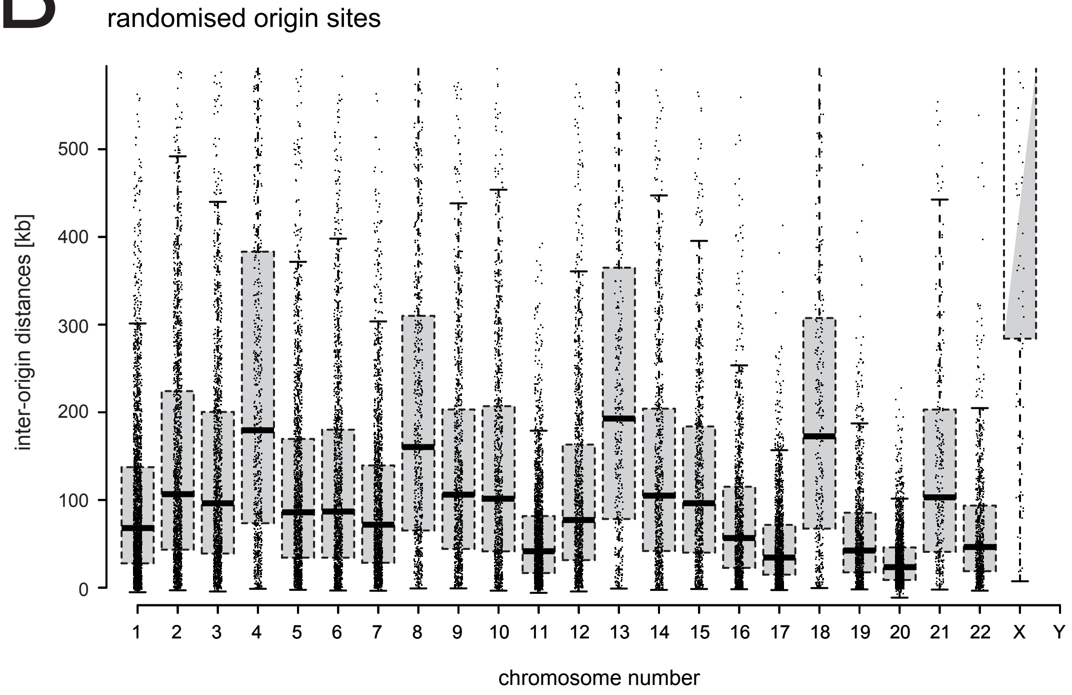
randomised origin sites
inter-origin distances [kb]
0
100
200
300
400
500
1 2 3 4 5 6 7 8 9 10 11 12 13 14 15 16 17 18 19 20 21 22 X Y
chromosome number

Figure S6

chromosome 17: 73,040 - 74,100kb

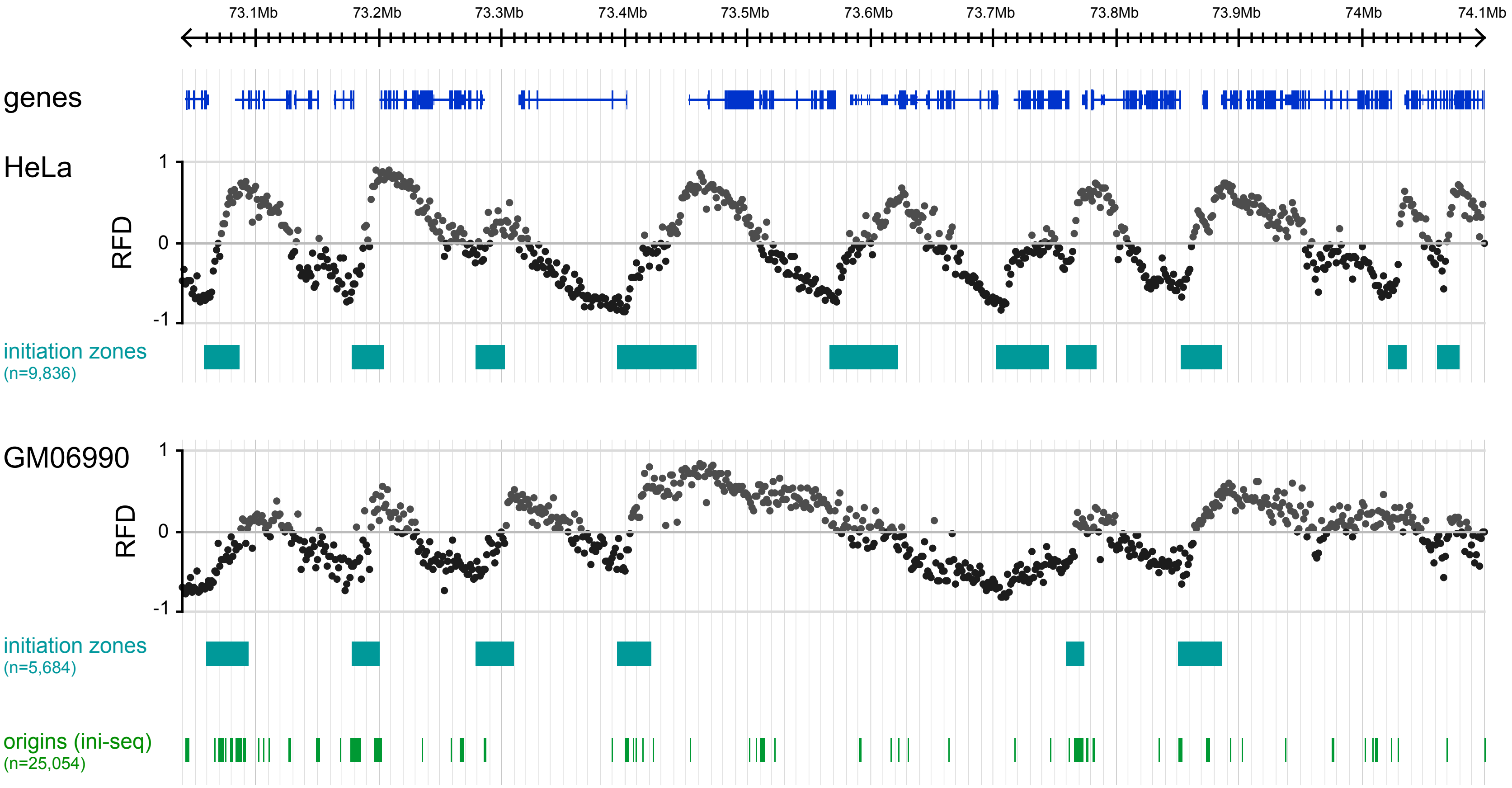

Figure S7

A

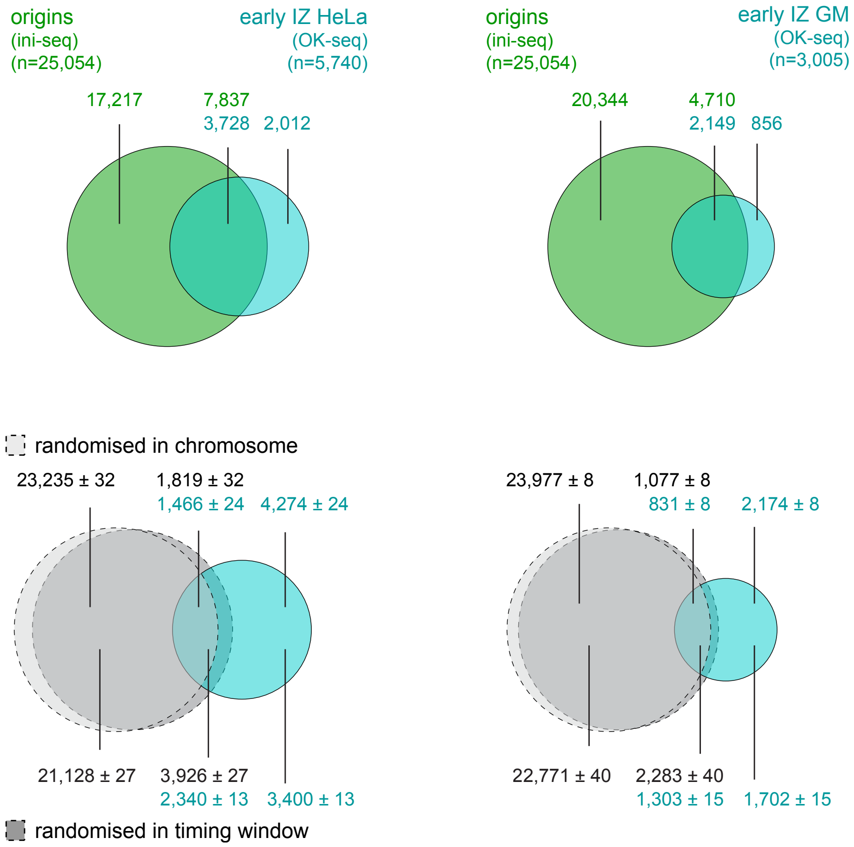
origins
(ini-seq)
(n=25,054)
early IZ HeLa
(OK-seq)
(n=5,740)
17,217
7,837
3,728
2,012
origins
(ini-seq)
(n=25,054)
early IZ GM
(OK-seq)
(n=3,005)
20,344
4,710
2,149
856
randomised in chromosome
23,235 ± 32
1,819 ± 32
1,466 ± 24
4,274 ± 24
21,128 ± 27
3,926 ± 27
2,340 ± 13
3,400 ± 13
randomised in timing window
23,977 ± 8
1,077 ± 8
831 ± 8
2,174 ± 8
22,771 ± 40
2,283 ± 40
1,303 ± 15
1,702 ± 15

B

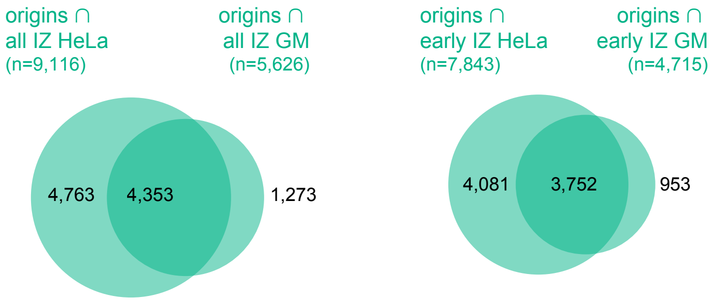
origins ∩
all IZ HeLa
(n=9,116)
origins ∩
all IZ GM
(n=5,626)
4,763
4,353
1,273
origins ∩
early IZ HeLa
(n=7,843)
origins ∩
early IZ GM
(n=4,715)
4,081
3,752
953

Figure S8

Table S1

Primer location	Primer sequence 5'–3'
MYC ori F	ACCAAGACCCCTTTAACTCAAGA
MYC ori R	CCTCGTCGCAGTAGAAATACG
MYC background site B F	GCCCCATCCCTTAGTGACAA
MYC background site B R	GTTTGCTGTCAGGCTTTTGG
MCM4 ori F	TTGGGTGGCTACTTGGTGTT
MCM4 ori R	GACCGCGTTTGTCGTTTTT
MCM4 background site B F	TCAAGAAGGCACTTTCGTATAAAT
MCM4 background site B R	AAGGCATGTCAGATTAGCAAAG
TOP1 ori F	CCTTATGCAAATCACAGCGGAG
TOP1 ori R	CGCTTTTAAACAACACGTCGG
TOP1 background site B F	CAAAATTGGGCTGTGAGGTTTT
TOP1 background site B R	TTTCCATACCATTAGCAGCAGG
Peak site P1 F	GACGCAGCTAGACCTTGGC
Peak site P1 R	CGACACTCACCCGCATCTC
Peak site P2 F	CCTCCCTTGGCTCTTACTGTTT
Peak site P2 R	GAGGTGCAGTGATAAACGAAGC
Peak site P3 F	GCGGATAGATACAGAAACGTAGG
Peak site P3 R	AAAAGTCTTGGGTGCAGGGTAT
Peak site P4 F	TTCTTTCGCTGAACACACGG
Peak site P4 R	GTTCCCGCCACCTATCCTC
Peak site P5 F	GGACTCAGACAAACCAAGAGC
Peak site P5 R	CATGGTAGTGTGCCCAAACA
Peak site P6 F	TGGACTCCTGTTGACTCACTC
Peak site P6 R	CACCTGTCGGCTTTGTTTCAT
Peak site P7 F	GGAAAGTGGATGGAAAGTGCAT
Peak site P7 R	GCCCAGCAATAGAAACGTTGAA
Peak site P8 F	ATCTCACAAGTCAGTCAGCCAA
Peak site P8 R	TTGTCACCCTGTCTGCGTC
Peak site P9 F	TAAGCACACCACTACTTGCCA
Peak site P9 R	ACAATTCAGAGACGAGGGGAC
Peak site P10 F	AGGCTGACACTTTCCCATCTT
Peak site P10 R	ACGTTCCTTAGGTGGTGGTTT
Peak site P11 F	GAGAACTCGGGGTCTGGC
Peak site P11 R	CGGCGTCAGTCTGGGATTG
Peak site P12 F	GGTGGGAAACGAAGATGAAAAGT
Peak site P12 R	CTAGTAAAATCTGTGGGGAAGCC
Peak site P13 F	GAATCCACATCCCCAGCAGAAG
Peak site P13 R	CTCGGGTAAGCGGGCTAG
Peak site P14 F	GAAGACGAGATGAGGGAGCTG
Peak site P14 R	CTACTCTCTCCCTGTTCCTGCT
Peak site P15 F	GCGTTCGTCACCAGCAATC
Peak site P15 R	ATTCATTCGGCAGCTATTTCGG
Peak site P16 F	CCCAGGATGCAGTTTGTGTC
Peak site P16 R	GCATTCCCTCGGCCATTTC
Background site B1 F	GGTGTTGTGCTTAGGTTGGAAC
Background site B1 R	TAGGCCGGACTCTCATACTTCA
Background site B2 F	CCTCTCAAGTACCCTCTGCAAT
Background site B2 R	TTTCATGCAACACCCAACACTT
Background site B3 F	TAGCTGCCCTTAAACACATTGC
Background site B3 R	TCAAAGAAGCTCAGGTATCCCC
Background site B4 F	ACCAGTTTCAGGATAAGGCTGT
Background site B4 R	GCTATCACCTGCTGTTTCTTGG
Background site B5 F	CCTCACGTCGTCTAACACCAAT
Background site B5 R	GAAGACACCAACCTGCCTGTAA
Background site B6 F	TTATGGGGATGGGGAAAGACAG
Background site B6 R	CACTGAGGGAGGGCTCTTAATT

Supplementary figure legends

Figure S1. Specificity of immunoprecipitation of nascent DNA. Nuclei from late G1 phase human EJ30 cells were incubated in a human cell-free DNA replication initiation system in the presence or absence of digoxigenin-dUTP. After fragmentation, DNA fragments were immunoprecipitated with non-conjugated agarose beads (mock IP) or anti-digoxigenin IgG-conjugated beads (dig IP). The amount of bead-associated DNA was quantified by the Qubit dsDNA HS Assay kit (Invitrogen). Data are expressed as proportions of 0.1% amount of input DNA, mean values of triplicate determinations are shown for one IP experiment.

Figure S2. Immunoprecipitation of nascent DNA. Nuclei from late G1 phase human HeLa cells were incubated in a human cell-free DNA replication initiation system, and nascent digoxigenin-labelled nascent DNA was isolated by immunoprecipitation with anti-digoxigenin IgG after fragmentation by sonification. The abundance of immunoprecipitated nascent DNA was quantitated by real-time PCR as detailed for Fig. 2A, using primer pairs located at the replication origins located near the promoters of the *MYC, MCM4* and *TOP1* loci, and at corresponding non-origin background sites (bg). Primer positions are shown in Fig. 2B.

Figure S3. Stringency of SICER peak calling. Peak calling and intersect analysis was performed by SICER excluding the input library for the indicated E-values. (A) Analysis of peak location for descending E-values. Analysis was performed for the HOX gene cluster locus on chromosome 7p15. Positions of reference genes and their transcription direction are indicated. Enrichment peaks called for immunoprecipitated DNA libraries A and B (IP-A and IP-B; light green) and the overlap between them (IP-A ∩ IP-B; dark green) are visualised on the integrated

genome viewer (IGV) for the indicated E-values. (A) Quantitative analysis of peak calling for descending E-values. Venn diagrams are shown for enrichment peaks called for libraries A and B (IP-A and IP-B) and the overlap between them (IP-A ∩ IP-B). Numbers of determined peaks are given for each E-value.

Figure S4. Ini-seq analysis of the laminB2 replication origin. Nascent DNA was isolated by immunoprecipitation and subjected to deep sequencing. The positions of sequencing reads are visualised on the integrated genome viewer (IGV) on a 40kb section of chromosome 19p13.3 (2.42-2.46Mb) for the total input control DNA (input) and two independent biological replicates of immunoprecipitated nascent DNA (IP-A and IP-B). Peaks of nascent DNA enrichment were called by SICER, both by integrating input DNA (w/i; blue bars), or not integrating input DNA (green bars). The areas of peaks present in both IP-A and IP-B (i.e. the intersect) are shown for both peak calling strategies (bottom row). Positions of reference genes and transcription direction are indicated. Positions of primer pair sites used for qPCR analyses reported previously (Keller et al., Nucl. Acids Res. 30, 2114-2123 (2002)) are indicated (black, origin sites; grey, background sites).

Figure S5. Comparison of origin peak widths and read counts between individual libraries. Origin peaks of immunoprecipitated nascent DNA were called by SICER excluding the input library. Data for immunoprecipitated nascent DNA libraries A (IP-A) and B (IP-B) are shown on the left and in the middle, respectively, while data for the overlap between them (IP-A ∩ IP-B) are shown on the right. (A) Distributions of peak widths for each human chromosome. (B) Distributions of accumulated sequencing read counts per peak for each human chromosome. Box and whisker plots without individual outliers are plotted, whiskers represent 1.5 times

the interquartile range above the upper quartile and below the lower quartile, and overall medians are indicated for each panel.

Figure S6. Distributions of inter-origin distances for the original (green) and a control distributions after randomisation within each chromosome (grey). Individual inter-origin distances are shown as scatter plots for each chromosome, overlaid on a box-and-whisker plot representation of the same data set. Only origin peaks present in both IP libraries (i.e. the intersects of IP-A and IP-B) were used. (A) Inter-origin distances of origin sites called by SICER at E=0.00001. (B) Inter-origin distances of the control distribution with randomised sites for each chromosome.

Figure S7. Comparison of origin sites identified by ini-seq with replication fork directionality profiles determined by Okazaki fragment sequencing (OK-seq). Raw data are plotted for replication fork directionality (RFD; forward strand for values >0, reverse strand for values <0) for HeLa and GM06990 cells, obtained from the OK-seq analysis by Petryk and co-workers, (http://157.136.54.88/cgi-bin/gbrowse/gbrowse/okazaki_ref/). Ascending segments in the RFD profiles representing DNA replication initiation zones are indicated by light blue bars for both cell lines. Positions of origins determined by ini-seq are indicated by green bars. The same genomic location is analysed as shown in Fig. 5a of the OK-seq analysis paper (Petryk et al., Nat. Commun. 7; doi: 10.1038/ncomms10208 (2016)).

Figure S8. Overlap analysis of origin sites identified by ini-seq with early replicating initiation zones identified by OK-seq. (A) Top row: Venn diagram analysis of replication origins determined by ini-seq (green) with early replicating initiation zones (IZ) determined by OK-seq (light blue) in HeLa (left) and GM06990 cells (right). Early replicating initiation zones were selected as the intersect of initiation zones

determined by OK-seq (Petryk et al., Nat. Commun. 7; doi: 10.1038/ncomms10208 (2016)) with the early replicating domain S1 taken from (Dellino et al., Genome Res. 23, 1-11 (2013)). Bottom row: overlap analysis of control sites after randomisation within each chromosome (dashed light grey) or within DNA replication timing windows (dashed dark grey) with early replicating initiation zones (light blue). Overlays of Venn diagrams are shown for these two analyses. Absolute numbers of origins/control sites and initiation zones are indicated for each section of the Venn diagrams. (B) Overlap analysis of origins detected by ini-seq present in genome-wide IZs (left) and early replicating IZs (right) in HeLa cells with those in GM06990 cells.

Table S1. PCR primers used in this study.

Table S2. Genomic coordinates of human DNA replication origins identified by ini-seq. Determination by SICER at E = 10e-1.

Table S3. Genomic coordinates of human DNA replication origins identified by ini-seq. Determination by SICER at E = 10e-5.
